# The persimmon (*Diospyros oleifera* Cheng) genome provides new insights into the inheritance of astringency and ancestral evolution

**DOI:** 10.1038/s41438-019-0227-2

**Published:** 2019-12-18

**Authors:** Qing-gang Zhu, Yang Xu, Yong Yang, Chang-fei Guan, Qiu-yun Zhang, Jing-wen Huang, Don Grierson, Kun-song Chen, Bang-chu Gong, Xue-ren Yin

**Affiliations:** 1 Zhejiang Provincial Key Laboratory of Horticultural Plant Integrative Biology, Zhejiang University, Zijingang Campus, Hangzhou, 310058 PR China; 2 State Agriculture Ministry Laboratory of Horticultural Plant Growth, Development and Quality Improvement, Zhejiang University, Zijingang Campus, Hangzhou, 310058 PR China; 3 Research Institute of Subtropical Forestry, Chinese Academy of Forestry, Hangzhou, 311400 PR China; 4 College of Horticulture, Northwest A&F University, Yangling, PR China; 5 Plant & Crop Sciences Division, School of Biosciences, University of Nottingham, Sutton Bonington Campus, Loughborough, UK

**Keywords:** Genome, Population genetics

## Abstract

Persimmon (*Diospyros kaki*) is an oriental perennial woody fruit tree whose popular fruit is produced and consumed worldwide. The persimmon fruit is unique because of the hyperaccumulation of proanthocyanidins during fruit development, causing the mature fruit of most cultivars to have an astringent taste. In this study, we obtained a chromosome-scale genome assembly for ‘Youshi’ (*Diospyros oleifera*, 2n = 2x = 30), the diploid species of persimmon, by integrating Illumina sequencing, single-molecule real-time sequencing, and high-throughput chromosome conformation capture techniques. The assembled *D. oleifera* genome consisted of 849.53 Mb, 94.14% (799.71 Mb) of which was assigned to 15 pseudochromosomes, and is the first assembled genome for any member of the Ebenaceae. Comparative genomic analysis revealed that the *D. oleifera* genome underwent an ancient γ whole-genome duplication event. We studied the potential genetic basis for astringency development (proanthocyanidin biosynthesis) and removal (proanthocyanidin insolublization). Proanthocyanidin biosynthesis genes were mainly distributed on chromosome 1, and the clustering of these genes is responsible for the genetic stability of astringency heredity. Genome-based RNA-seq identified deastringency genes, and promoter analysis showed that most of their promoters contained large numbers of low oxygen-responsive motifs, which is consistent with the efficient industrial application of high CO_2_ treatment to remove astringency. Using the *D. oleifera* genome as the reference, SLAF-seq indicated that ‘Youshi’ is one of the ancestors of the cultivated persimmon (2n = 6x = 90). Our study provides significant insights into the genetic basis of persimmon evolution and the development and removal astringency, and it will facilitate the improvement of the breeding of persimmon fruit.

## Introduction

*Diospyros* L. belongs to the Ebenaceae, a plant genus that includes over 500 species that are distributed worldwide^1^ and is one of the largest angiosperm genera^2^. Some of its species (e.g., *Diospyros kaki* Thunb) produce edible fruit, and others are sources of ebony wood, stock, medicinal materials or ornamental plants^3^. The most well-known species is *Diospyros kaki*, which originated from China^4^. One fossil leaf of wild persimmon (*Diospyros miokaki*) that is ~25 million years old was found in the Shandong Province of China and stored in the Shanwang Paleontological Museum^5^. In ancient China, persimmon was first described in ‘Li Ji·Nei Ze’ (*circa* 450 BC, written by Ji Kong, grandson of Confucius), and it began to be considered an important food during the Warring States Period (475–221 B.C.)^6^. The persimmon was widely cultivated in China during the Tang Dynasty (618–907 A.D.) due to the development of grafting and technological approaches for removing astringency, and it was introduced to Japan and spread to the Korean peninsula in the 15th century and to Europe and then America in the 18th–20th centuries^3^. Persimmon has been cultivated mainly in oriental countries (e.g., China, Japan, and Korea) but is widely distributed in other areas (e.g., China, Japan, and Korea). Australia, Brazil, Israel, Spain, and the United States)^7^. The most recent statistical data indicate that persimmon fruit production in China has reached 400 million tons annually (FAO, 2016).

Persimmon fruit are unique in their accumulation of proanthocyanidins (PAs, also known as condensed tannins, CTs), and soluble CTs (SCTs) cause fruit astringency in most cultivars, even at commercial maturity^8,9^, reducing consumer appeal. Thus, persimmon is a characteristic material for studying PA synthesis, accumulation and metabolism in fruit. PAs also prevent benefits such as strong oxidation resistance, and they are widely used in cosmetics as antioxidants and anti-aging ingredients^10^. PAs are also widely used in wastewater treatment because of their scavenging ability towards radioactive and heavy metals^10–14^. The biosynthesis of PAs is controlled by a series of structural genes in the shikimate pathway^15^; however, the molecular mechanisms and evolution of PA accumulation in persimmon remain elusive^16^. In addition to the study of PA metabolism, in recent years, persimmon has been used as a model to understand fruit anaerobic environment interactions^17,18^ and the evolution of sex chromosome systems in higher plants^19^. However, most of these studies were conducted based on the de novo assembly of transcriptomic sequences due to the absence of complete persimmon genome sequences for either *Diospyros* species or other Ebenaceae. This deficiency has inhibited research on evolution and heredity in persimmon and other plants in the Ebenales.

Within *Diospyros*, there are three main species cultivated for fruit production: *Diospyros lotus*, *Diospyros oleifera*, and *Diospyros kaki*, and all three of which produce astringent fruit. *D. kaki* is hexaploid (2n = 6x = 90), while *D. lotus* and *D. oleifera* are diploid (2n = 2x = 30)^20^. *D. kaki* accounts for the greatest number of commercial fruit tree cultivars, and *D. lotus* is widely used as a rootstock. Compared to *D. kaki* and *D. lotus*, *D. oleifera* exhibits two additional characteristics: the generation of trichomes and the excretion of oils on the fruit surface (Supplementary Fig. 1). Here, the material ‘Youshi’ (the traditional common name in China for *D. oleifera* Cheng) was used; Youshi means ‘oil persimmon’ in Chinese. Another Ebenales plant, avocado (*Persea americana*), is also well known for containing high levels of lipids^21^. Fu et al.^22^ sequenced the complete chloroplast genomes from *D. kaki*, *D. lotus*, *D. oleifera*, *D. glaucifolia* and *D*. ‘Jinzaoshi’ and found that *D. kaki* was more closely related to *D. oleifera*, whereas *D. lotus* exhibited a closer relationship with *D. glaucifolia*. Kanzaki^4^ proposed that the commercialized persimmon (*D. Kaki*) originated from southern China through polyploidization from diploid ancestors. The complete genome sequence for *Diospyros* would be tremendously useful for clarifying these issues.

In this study, *D. oleifera* (‘Youshi’) was chosen as the material for genome sequencing. The PacBio RSII and HiSeq sequencing techniques were used to generate a high-quality genome assembly and annotation for this diploid persimmon. Due to the importance of astringency for the persimmon industry, the genetic basis for astringency development and removal was also investigated. Based on the obtained genome sequence, the first genetic linkage map was constructed for persimmon using SLAF-seq, which provided information about the evolution of persimmon.

## Results

### Genome assembly and annotation

The ‘Youshi’ genome was sequenced using the Illumina HiSeq (Illumina, USA) and PacBio Sequel platforms, and the assembled scaffolds were ordered using the Hi-C technique. The detailed assembly process is illustrated in Supplementary Fig. 2. The project generated ~86 gigabases (Gb) of high-quality sequences (Supplementary Tables 1 and 2), representing ~100× coverage of this diploid ‘Youshi’ persimmon genome. The final genome assembly was 849.53 Mb in size, very close to the predicted size of 853.3 Mb based on nuclear weight measurements performed via flow cytometry (Supplementary Fig. 3), and it consisted of 4728 scaffolds (≥1 kb) with an N50 of 42.43 Mb and 5919 contigs (≥1 kb) with an N50 of 890.84 kb (Supplementary Table 3). The GC content of the assembled persimmon genome was 37.40% (Table 1). Notably, 94.14% (799.71 Mb) of the genome was anchored to 15 pseudochromosomes (Supplementary Table 4), and a total of 556.36 Mb (64.96%, Supplementary Table 5) of repetitive sequences were identified.

**Table. 1:** Statistics of persimmon genome assembly and annotation.

Genome size (Mb)	849.53
Total size of assembled scaffolds (Mb)	856.414
Number of scaffolds (≥1 Kb)	4728
N50 scaffold length (Mb)	1.42
Longest scaffold (Mb)	57.59
Total size of assembled contigs (Mb)	856.35
Number of contigs (≥1 kb)	5919
N50 contig length (kb)	890.84
Largest contig (kb)	9384.04
GC content (%)	37.40
Number of gene models	32516
Gene length (Mb)	220.26
Mean gene length (bp)	6773.92
Total exon length (Mb)	41.58
Mean exon length (bp)	251.99
Total intron length (Mb)	178.68
Mean intron length (bp)	1082.91

The quality of the assembly was further assessed by three independent methods. First, it was verified that it contained a majority of the core eukaryotic genes (98.03% and 77.02%, respectively, Supplementary Table 6) and genes in the BUSCO (Benchmarking Universal Single-Copy Orthologs) datasets^23^ (89.86%, Supplementary Table 7). Second, the high-throughput sequencing (Illumina HiSeq) data were aligned to the genome sequence using BWA software^24^^,^ and the results indicated that more than 98.58% of the sequences could be mapped to the assembled genome (Supplementary Table 8). Third, a Hi-C intrachromosomal contact map suggested that all bins could be allocated to 15 pseudochromosomes (Supplementary Fig. 4). Thus, all of these results support the conclusion that this assembled ‘Youshi’ persimmon genome is of high quality at the chromosome scale.

**Fig. 1:**
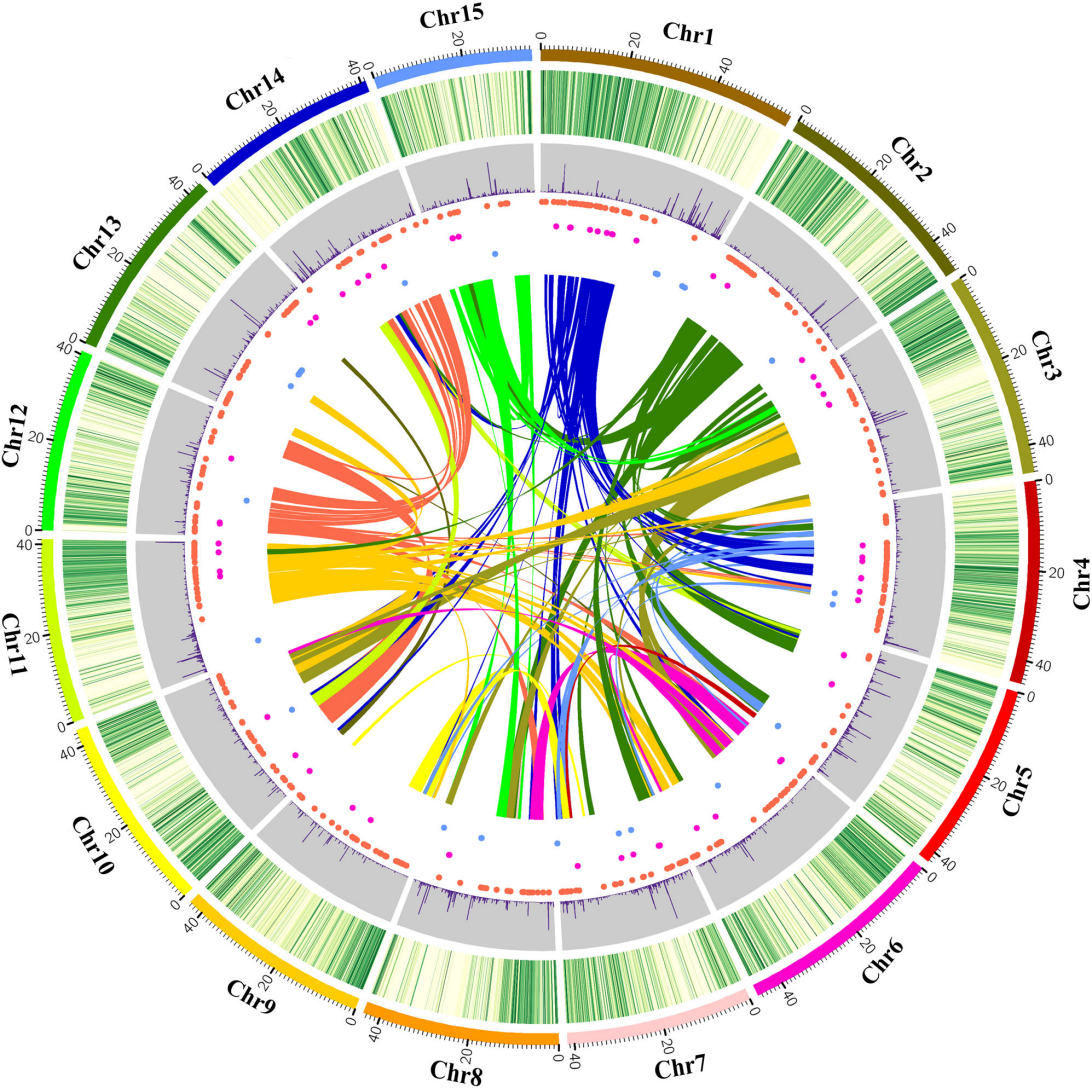
Homologous genome blocks in persimmon. Chr1-15 are pseudochromosomes. The first (green) circle shows the gene density on chromosomes, and the second (purple) circle shows the pseudogene density. The brown, purple and blue dots show the distribution of tRNAs, miRNAs, and rRNAs, respectively. The inner lines represent the relationships between syntenic blocks. Each line represents a syntenic block.

A total of 32,516 putative genes were predicted, with an average gene length of 6773 bp; 80.53% of these genes shared homology with known genes, and 95.95% of these genes were functionally annotated (Supplementary Tables 9 and 10; Supplementary Fig. 5). Among these predicted genes, only 25,379 (78.05%) could be anchored to the 15 pseudochromosomes (Supplementary Fig. 6). A total of 1540 noncoding RNAs, including miRNAs, rRNAs, and tRNAs, were identified by in silico prediction (Supplementary Table 11). In addition, 4381 pseudogenes were predicted in the persimmon genome (Supplementary Table 12). Motif and domain annotation analysis based on the amino acid sequences of 32,516 predicted genes indicated a total of 2802 motifs and 36,198 domains (Supplementary Table 13). There were 137 gene syntenic blocks and 3612 paralogous gene groups identified based on the self-alignment of the 25,379 chromosome-anchored genes, indicating that the persimmon genome has undergone frequent interchromosome fusions and segmental duplications during its evolutionary history (Fig. 1).

### Comparative genomic and genome evolutionary analysis

**Fig. 2:**
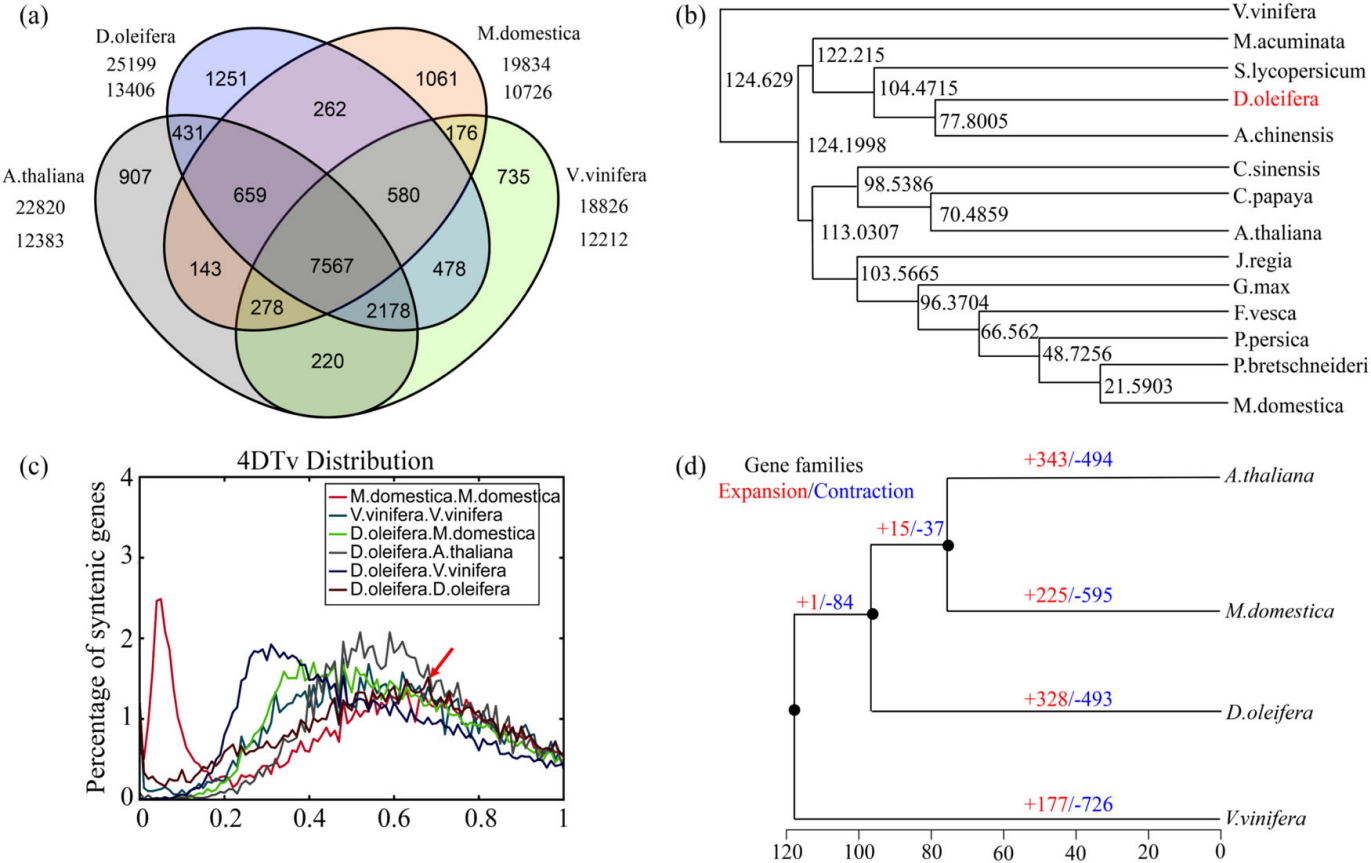
Comparative genomic analysis of persimmon and other species. **a** Venn diagram of shared orthologous gene families in persimmon, *Arabidopsis thaliana*, *Malus domestica* and *Vitis vinifera*. The number of gene families is listed for each component. **b** Phylogenetic tree constructed from persimmon single-copy gene families with 13 other species, including *Vitis vinifera* (Vvinifera_145_Genoscope), *Actinidia chinensis* (v1.0), *Musa acuminata*, *Solanum lycopersicum* (SL2.40), *Diospyros oleifera*, *Citrus sinensis*, *Glycine max* (Soybean_Williams82), *Carica papaya*, *Arabidopsis thaliana* (TAIR10), *Juglans regia*, *Fragaria vesca*, *Prunus persica* (v2.0), *Pyrus bretschneideri*, and *Malus domestica* (v1.0). The numbers indicate the divergence time. **c** Distribution of 4DTv distance between syntenic orthologous genes. The red arrow indicates the peak value for persimmon. **d** Gene family expansion and contraction analysis. Gene family expansions and contractions are indicated by numbers in red and blue, respectively. The gray areas represent the conserved gene families.

A gene family cluster analysis of the complete gene sets of persimmon (*D. oleifera*), apple (*M. domestica*), Arabidopsis (*A. thaliana*) and grape (*V. vinifera*) was performed. A total of 25,199 gene families in the persimmon genome were grouped into 13,406 gene clusters, with 7567 gene clusters being shared by all four species (Fig. 2a). Furthermore, 3644 persimmon-specific genes in 1251 clusters were identified (Supplementary Table 14), which are annotated in Supplementary Table 15. An expanded analysis was performed with 13 other sequenced plant genomes, and single-copy genes were used for phylogenetic tree construction. The results showed that *D. oleifera* is relatively closely related to *Actinidia chinensis* (kiwifruit) and *Solanum lycopersicum* (tomato) (Fig. 2b). The phylogenetic tree also indicated that *D. oleifera* diverged phylogenetically from *A. chinensis* approximately 77.80 million years ago (Mya), after the divergence of *S. lycopersicum* at 104.47 Mya (Fig. 2b).

The 4DTv (fourfold synonymous third-codon transversion) value peaked at ~1.51 for *D. oleifera* (Fig. 2c), indicating that only one ancient γ whole-genome duplication (WGD) occurred in the *D. oleifera* genome lineage, which was further confirmed by ks analysis (Supplementary Fig. 7). There were 328 and 493 gene families (annotations are listed in Supplementary Table 16) showing expansion or contraction, respectively, after divergence from kiwifruit (Fig. 2d), suggesting that more *D. oleifera* gene families have experienced contraction than expansion during adaptive evolution.

A total of 414 and 3637 genes in the expanded families were annotated (Supplementary Tables 17 and 18) to kyoto encyclopedia of genes and genomes (KEGG) pathways and gene ontology (GO) terms, respectively. KEGG analysis showed that most of the expanded genes were involved in plant-pathogen interactions, starch and sucrose metabolism and phenylpropanoid biosynthesis. GO analysis revealed that in addition to primary metabolic processes, the expanded orthogroups were involved in the defense response, sexual reproduction and flavonoid biosynthetic processes. Forty-seven of the genes from contracted gene families were clustered in 28 KEGG pathways, including plant hormone signal transduction, sesquiterpenoid, and triterpenoid biosynthesis and beta-alanine metabolism (Supplementary Table 19). The GO terms of the genes from contracted gene families were mainly related to oxidation-reduction processes, protein phosphorylation and pentacyclic triterpenoid biosynthetic processes (Supplementary Table 20). The functional annotations of the genes in expanded and contracted gene families highlighted various traits of persimmon, including its high contents of sugar and flavonoids, dioecy, low levels of terpenes, and strong adaptability.

### Characterization of putative genes in proanthocyanidin biosynthesis pathways

**Fig. 3:**
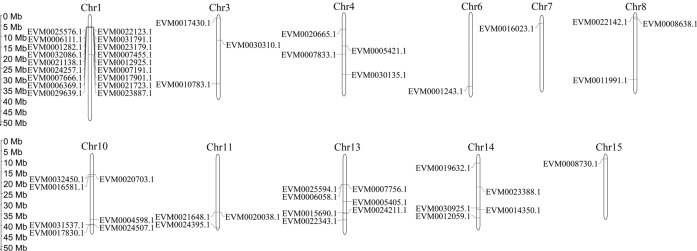
Genome location of proanthocyanidin biosynthesis genes in the persimmon genome.

A unique feature of persimmon is that it accumulates a high content of PAs during fruit development. In the genome, we found 57 genes involved in PA biosynthesis (Supplementary Table 20), all of which were mapped to the *D. oleifera* genome. The results indicated that 33.96% of the genes involved in PA biosynthesis were located on chromosome 1 (Fig. 3) and formed a gene cluster enriched in these genes (Supplementary Fig. 8).

### Genome-wide RNA-seq analysis of astringency removal in persimmon fruit by artificial high CO_2_ treatment

Most commercialized persimmon cultivars are of the astringent type; thus, understanding PA biosynthesis (astringency formation) and removal (deastringency) is important. A few candidate genes related to alcoholic fermentation and transcription factors that regulate their expression have been previously identified and shown to contribute to astringency removal^9,17,18^. However, because of the absence of a complete genome sequence, many of the genes potentially involved in astringency removal were not identified.

**Fig. 4:**
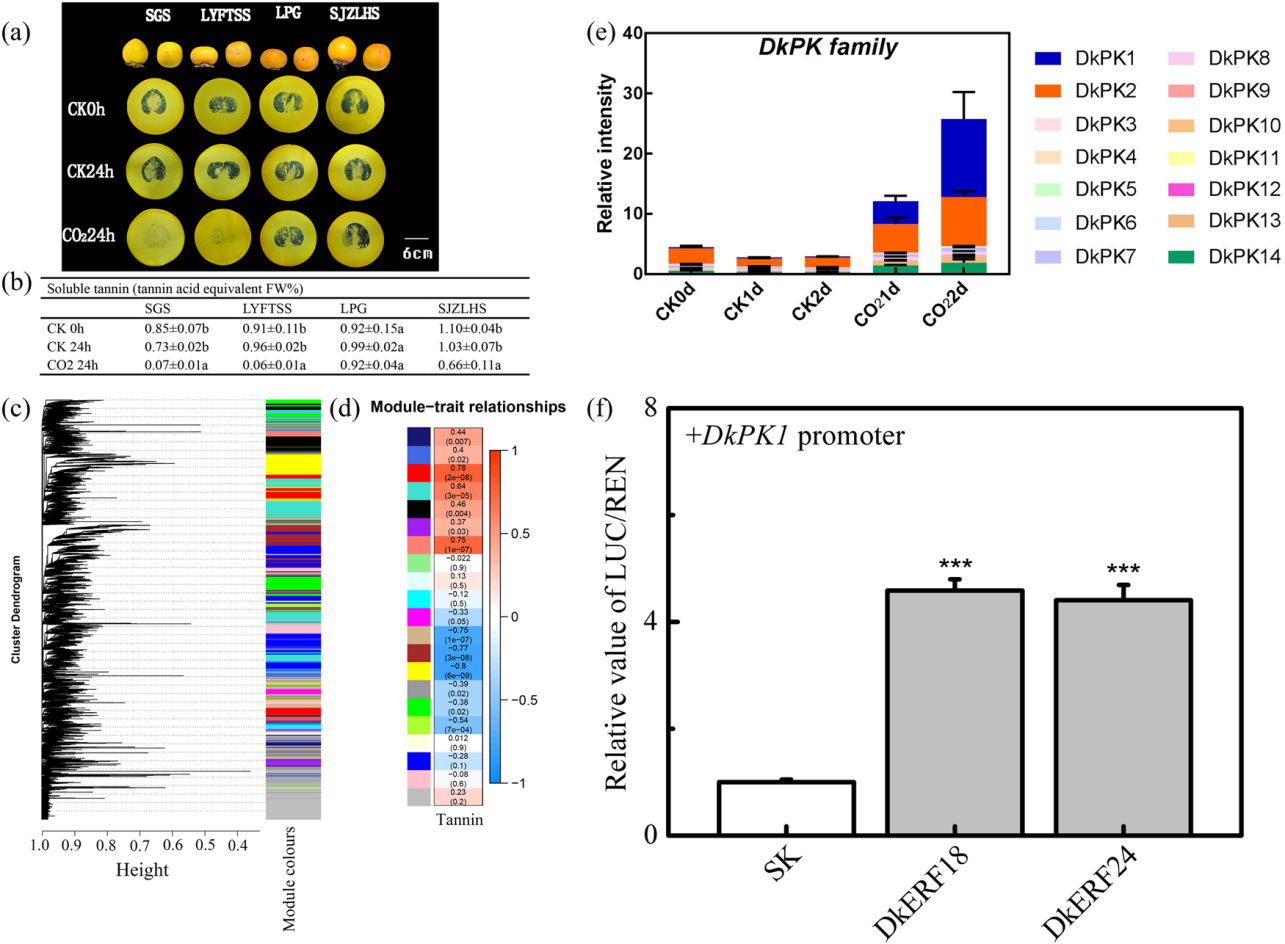
Persimmon fruit deastringency analysis and WGCNA analysis. **a** Tannin printing of CO_2_-treated persimmon fruit at 1 day in storage. The intensity of black reflects the soluble tannin content. The fruit were treated with high CO_2_ (95% CO_2_, 4% N_2_, 1% O_2_) and air (control) within airtight containers for 1 day at 20 °C. **b** Effects of CO_2_ treatment on soluble tannin contents in four selected cultivars of persimmon fruit. Different letters represent significant differences (*P* < 0.05). **c** WGCNA dendrogram indicating the expression of different gene modules in all 36 persimmon samples. **d** Trait and module relationship analysis. Different colors represent different modules. **e** Expression patterns of PK family genes in ‘Jingmianshi’ fruit treated with high CO_2_ (95% CO_2_ + 4% N_2_ + 1% O_2_). **f** Regulatory effects of DkERF18 and DkERF24 on the DkPK1 promoter.

High CO_2_ treatment (95% CO_2_ + 4% N_2_ + 1% O_2_) is the most effective method for tannin removal. This treatment results in elevated ADH and PDC activities and triggers acetaldehyde metabolism^9,18,25,26^. The acetaldehyde that is produced converts the soluble condensed tannins into insoluble condensed tannins^27,28^, so that they no longer contribute to astringency. Here, 53 astringent-type cultivars of persimmon fruit (listed in Supplementary Table 22) were collected and subjected to high CO_2_ treatment for 24 h. Four representative cultivars, two showing rapid deastringency after treatment (*D. kaki* cv. Sigoushi, SGS and cv. Luoyangfangtianshengshi, LYFTSS) and two showing slow deastringency (*D. kaki* cv. Laopige, LPG and cv. Shijiazhuanglianhuashi, SJZLHS), were selected according to quantitative soluble tannin analysis. After high CO_2_ treatment, SGS and LYFTSS exhibited lower soluble tannin contents (<0.1 FW%), while LPG and SJZLHS exhibited higher soluble tannin contents (0.92 and 0.66 FW%, respectively) (Fig. 4b).

When these four cultivars were used for RNA-seq, the differentially expressed genes (DEGs) were clustered into 21 coexpression modules (Fig. 4c) through WGCNA (weighted gene coexpression network analysis), and tannin contents were correlated with the module eigengenes (Fig. 4d). Three significantly negative correlation modules (<−0.6) were found (tan, −0.75; brown, −0.77; yellow, −0.64), and the genes within these modules are listed in Supplementary Table 23. As expected, genome-wide transcriptomic analysis provided a more comprehensive overview of astringency removal. The previously characterized genes related to alcoholic fermentation, *DkPDC1* (EVM0027273), *DkPDC2* (EVM0022732), and *DkADH1* (EVM0007501), were simultaneously clustered in the brown or yellow module. Moreover, a key enzyme that catalyzes the phosphoenolpyruvate (PEP)-to-pyruvate transition under low-oxygen conditions (Supplementary Fig. 9 ^29^), the pyruvate kinase (PK) gene (EVM0008535, *DkPK1*), was found to be one of the hub genes (Supplementary Fig. 10). The expression in fruit of members of the *DkPK* family was verified in ‘Jingmianshi’ fruit (*D. kaki*, astringent type), and the transcript levels of *DkPK1* were found to be higher than those of any other members of this gene family (Fig. 4e). Furthermore, dual-luciferase assays indicated that two previously characterized hypoxia/high-CO_2_-responsive transcription factors (*DkERF18* and *DkERF24*) could trans-activate the promoter of *DkPK1* (Fig. 4f).

To understand the genetic basis of the failure of astringency removal during fruit development in astringent-type persimmons, the putative promoter regions (−2000 bp) of all of the previously characterized hypoxia/high-CO_2_-responsive transcription factors (Supplementary Table 24) and candidate genes included in Supplementary Fig. 9 were obtained (Supplementary Fig. 11 and 12). Motif analysis indicated that anaerobic-responsive elements (GC-rich motif: GCC[C/G]C, GT-rich motif: GGTTT)^30^ were present in all of these promoters (Supplementary Figs. 11 and 12). Each of these genes exhibited more than one anaerobic-responsive element within a single promoter (Supplementary Figs. 11 and 12), and these results confirm the genetic and mechanistic basis of the effectiveness of low oxygen (resulting from high CO_2_ treatment) in causing astringency removal.

### *D. oleifera* genome-based genetic map analysis

**Fig. 5:**
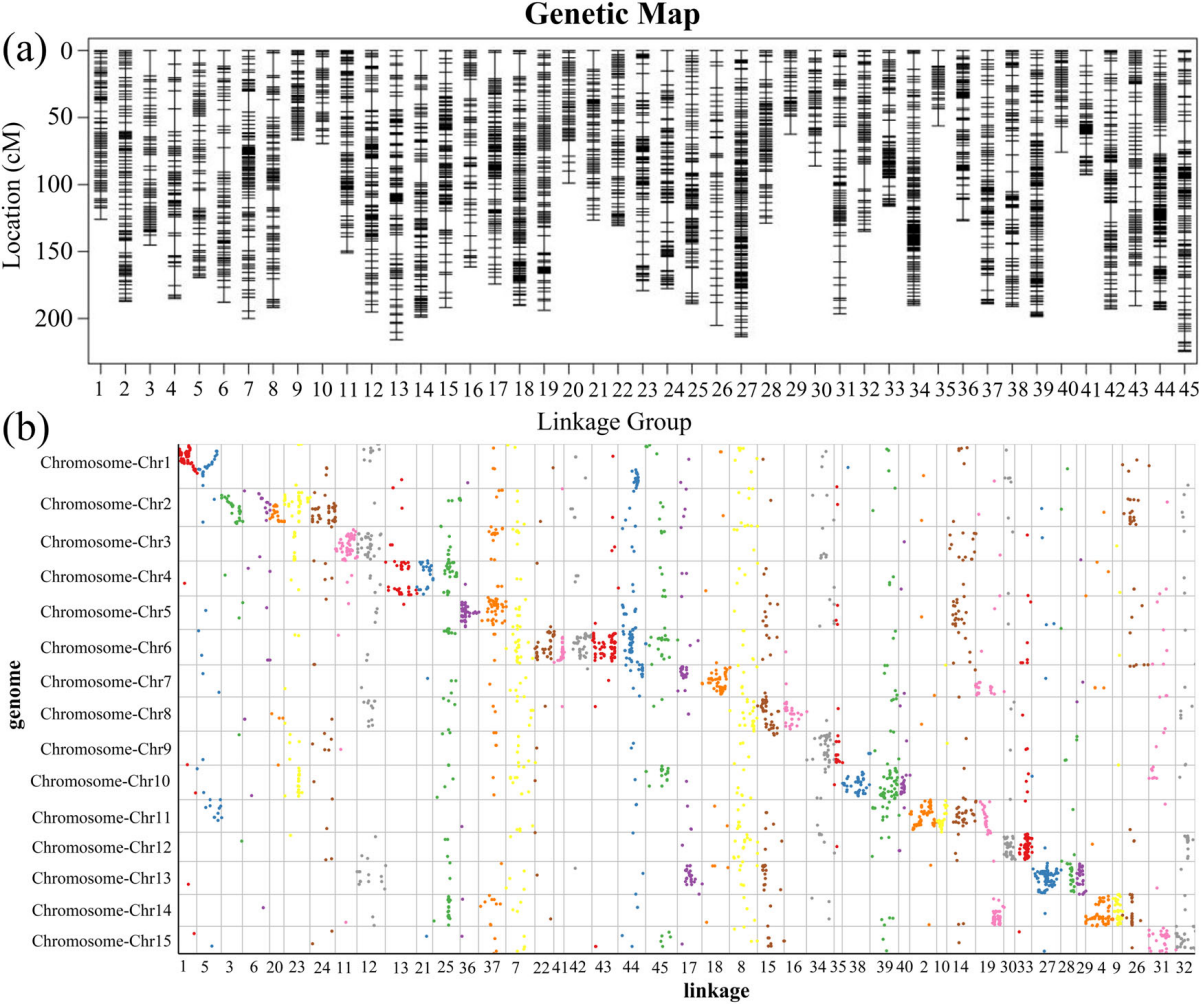
Genetic map analysis of persimmon. **a** High-density genetic map of *D. kaki*. **b** Correlation between the genetic and physical positions of the SLAF markers. *D. kaki* represents the genetic group, and the *D. oleifera* genome represents the physical position.

Two commercial persimmon cultivars, ‘Taishuu’ *(Diospyros kaki* cv. Taishuu, male) and ‘Luotiantianshi’ (*Diospyros kaki* cv. Luotiantianshi, female), were used as parents for hybridization. Due to the high rate of abortion in hybrid seeds, even after using the embryo rescue culture technique^31^, only 77 F1 progeny were obtained. Then, an SLAF-seq (specific-locus amplified fragment sequencing) library was constructed for the two persimmon parents and their F1 offspring. The evaluation of the *D. kaki* SLAF DNA library indicated that the efficiency of *Rsa*I and *Hae*III digestion was 86.80%. The percentage of the mapping of the paired-end reads in the control (*Oryza sativa* L. japonica) was 87.92%. The average sequencing depth of the SLAF markers was 20.40-fold (Supplementary Table 25). After filtering, for the F1 population, 20,405 SLAFs with three segregation patterns were used for genetic map construction, and 11,204 markers were used (Supplementary Table 26). After linkage analysis, 11,204 markers were anchored to 45 high-density linkage groups of *D. kaki*, with a minimum LOD score (MLOD) of 7.0. The final map was 7168.74 cM long, with an average intermarker distance of 0.64 cM (Supplementary Table 27). The largest LG (linkage group) was LG45, which contained 222 markers with a length of 224.67 cM; the smallest was LG35, which contained 118 markers that spanned 56.23 cM (Supplementary Table 27). The percentage of gaps ≤5 cM between adjacent markers of the 45 LGs ranged from 92.74 to 99.35% (average of 97.13%), and the largest gap was 19.77 cM in LG17 (Fig. 5a). After correcting genotyping errors using SMOOTH algorithms, 1140 segregation distortion markers with *P* < 0.01 were retained to increase genomic coverage in the final genetic map, which was further evaluated by the haplotype (Supplementary Fig. 13) and heat maps (Supplementary Fig. 14).

Based on the *D. oleifera* genome, the colinear relationship between the *D. kaki* genetic map and the *D. oleifera* genome was analyzed. The SLAF sequences of the 11,204 mapped markers were aligned to the genomes using BLASTN analysis with an e-value cutoff of 1e−5, revealing that 26.86% (*n* = 3009) of the markers were assigned to the *D. oleifera* genome (Supplementary Table 20). Moreover, 45 LGs were clearly assigned to the 15 chromosomes (Fig. 5b and Supplementary Table 28). Different numbers of LGs were assigned to different chromosomes, such as chromosomes 1/3/5/7/9/12/15, each with two LGs, chromosomes 4/8/10/13/14 were each assigned three LGs, and four, five and seven LGs were assigned to chromosomes 11, 2 and 6, respectively (Fig. 5b). Using the Spearman correlation coefficient approach, the mapped markers (*n* = 3009) of the *D. kaki* genetic map were found to show significant collinearity with the *D. oleifera* genome, and the correlation between the genetic and physical positions of the SLAF markers is shown in Fig. 5b.

## Discussion

Persimmon is a widely grown woody tree, and its fruit are valued by consumers because of their high concentrations of antioxidant compounds (especially PAs) and edible fiber, and they serve as a primary source of medicines^3,32,33^. Although persimmon is economically and culturally important in oriental countries, its genome sequence has not been published. Here, using PacBio and Hi-C technology, the genome sequence of persimmon was assembled from diploid ‘Youshi’ (*D. oleifera*), which provides a basic resource for the future persimmon research and industrial applications. A genome sequence of 849.53 Mb was assembled with a contig N50 of 0.89 M, and 94.14% of the genome was anchored to 15 pseudochromosomes. The quality of the assembly was further assessed by using core genetic integrity assessment and RNA-seq data. There were 32,516 predicted gene locations in the persimmon genome, which is similar to results from other plant species, such as tomato (*N* = 34,879)^34^ and pomegranate (*N* = 30,903)^35^. A total of 556.36 Mb (64.96%, Table S7) of repeat sequences were identified, which is similar to the amount in sorghum (62%)^36^ and higher than that in apple (42%)^37^, grapevine (41.4%)^38^ and rice and *Arabidopsis* (14 and 17%, respectively)^39,40^. Additionally, a total of 4012 simple sequence repeats were evenly distributed across the pseudochromosomes, which are beneficial for molecular breeding (Supplementary Table 5). The obtained *D. oleifera* genome not only provides basic information for persimmon research and utilization but also represents the first reported complete genome for Ebenales. Thus, the *D. oleifera* genome could be further used for better understanding genome evolution and the relationships of plants in Ebenales.

The phylogenetic tree compiled from the whole-genome analyses showed that *D. oleifera* diverged from kiwifruit approximately 77.80 Mya, ago and the estimated times of the separation from the lineages with *A. thaliana* and *V. vinifera* were ~124.20 and 124.63 years ago, respectively. Following the ancient hexaploidization event (γ) shared by core eudicots, including kiwifruit, there were two additional independent WGD events^41^. For *D. oleifera*, the 4DTv analysis indicated that persimmon underwent an ancient γ WGD event. Whether this WGD event is shared by other members of Ebenales will have to be determined through further analysis when more Ebenales genome sequences become available.

The KEGG and GO analyses indicated that the expanded gene families are mainly involved in starch and sucrose metabolism, which may have conferred greater attractiveness on the persimmon fruit during evolution. Some other expanded genes were found to participate in phenylpropanoid biosynthesis, flavonoid biosynthesis, and anthocyanin biosynthesis, and all of these pathways, as well as PA biosynthesis, are derived from the shikimate pathway^15^. The phenylpropanoids are considered to be key mediators involved in plant resistance to biotic and abiotic stress responses^42^. These expanded gene families are responsible for the accumulation of PAs in persimmon but also cause fruit astringency, which is not desirable to humans and unexpected animal and insect invasions. In recent years, a non-astringent persimmon that originated from spontaneous mutants of astringent persimmon has increased in importance in fruit industry^7^. Experiments indicate that astringent persimmons are more resistant to cold than non-astringent persimmons^3^. Thus, the expansion of the relevant gene families may have provided an important way to generate abiotic and biotic resistance in persimmon, which would have been critical for the evolution of persimmon and the expansion of its cultivation.

The characteristics of *D. kaki* persimmon and ‘Youshi’ (*D. oleifera*) related to astringency development and removal were selected for further genome-based investigation. The fruit of hexaploid persimmon cultivars such as ‘Youshi’ also exhibit active PA biosynthesis. The basis for this characteristic was investigated via the genome-wide analysis of PA biosynthesis genes. Most of the PA biosynthesis genes were located on chromosome 1, where they formed a gene cluster. In the plant genome, it is common to find highly homologous genes involved in secondary metabolite synthesis located in a cluster on a chromosome^43^. These gene clusters may originate from duplication and divergence, and clustering may also improve gene function. Furthermore, related traits are likely to be stably inherited, and the genes in clusters could be regulated simultaneously. Most metabolites synthesized by gene clusters are defense or tolerance related^44–47^. Arabidopsis, apple, and grape are predicted to exhibit 18, 73, and 23 genes involved in PA biosynthesis, respectively, but these genes are widely distributed on different chromosomes^37–39^. Thus, the clustering of PA biosynthesis genes provides the genetic basis for high PA content in persimmon. On the other hand, genome-based RNA-seq provided new insights regarding astringency removal by identifying the previously characterized *PDC* and *ADH* genes^9^ and new hypoxia-responsive genes (e.g., *DkPK1* and its transcriptional regulation). In *Arabidopsis*, a genome-wide investigation in seedlings predicted 49 core hypoxia-responsive genes^48^. Here, the assembled genome allowed the rapid searching of low-oxygen-responsive motifs in the putative promoter regions of these candidate genes. Our findings suggested that all of the candidate genes exhibited multiple low-oxygen-responsive motifs in their promoters (Supplementary Figs. 10 and 11), which provided genetic evidence of their role and effectiveness in postharvest astringency loss in response to a low oxygen environment.

High-density linkage maps are the basis for QTL fine mapping of traits, map-based gene cloning and marker-assisted breeding^49–51^. In persimmon, germplasm genetic diversity and marker-trait associations have been explored using different types of molecular markers^52–56^. However, none of this research has led to the construction of a high-quality genetic map for persimmon or for any member of the *Diospyros* genus, due to the limited number of hybrid offspring produced because of the abortion of hybrid seeds. SLAF-seq provides an economical and efficient method for the linkage mapping of nonmodel species with complex genomes^57^. Here, we obtained 77 F1 offspring from ‘Taishuu’ × ‘Luotiantianshi’ via the embryo rescue culture technique^31^ and constructed the first *D. kaki* genetic linkage map, with 11,204 markers spanning 7,168.74 cM. The average intermarker distance was 0.64 cM, which is comparable to the resolution reported for high-density linkage maps of *Vigna unguiculata* (0.35 cM)^58^, *Arachis hypogaea* (0.58 cM;^51^ 0.7 cM^59^), *Pleurotus tuoliensis* (1.0 cM)^50^ and *Gossypium barbadense* (1.09 cM)^49^. This genetic map should be useful for persimmon breeding as a genome reference and for establishing marker-trait associations, providing a basis for understanding the persimmon genome and its inheritance. These markers will be useful for screening important biological properties of persimmon, especially related to the accumulation of tannins and other factors affecting taste.

As the genome of hexaploid cultivated persimmon remains unpublished, the availability of the *D. oleifera* genome provides an alternative comparable reference for *D. kaki*. Based on the *D. oleifera* genome, 26.86% of the 11,204 markers (*n* = 3009) in the *D. kaki* genetic linkage map were assigned to and showed high colinearity with the newly completed *D. oleifera* genome sequences. Thus, it can be proposed that *D. oleifera* was one of the diploid ancestors of hexaploid *D. kaki*, and this evidence supports the previous hypothesis of Kanzaki^4^ and the preliminary predictions resulting from genomic in situ hybridization (GISH) studies^60^ and the comparison of chloroplast genome sequences^22,61^. The relationship between chromosomes and LGs suggested that *D. oleifera* may also be closely related to other ancestors of *D. kaki*, and chromosome breakage and rearrangement may have occurred during the evolution of *D. kaki* from *D. oleifera*.

In summary, the first genome of Ebenaceae, from *D. oleifera*, was sequenced and found to be approximately 849.53 Mb in size (N50 > 0.8 M), with 94.14% (799.71 Mb) of the sequences anchored onto 15 pseudochromosomes. Comparative genomics analysis revealed the genetic basis of astringency development (PA synthesis) and removal in persimmon via precipitation, caused by the production of acetaldehyde during the low-oxygen response. Furthermore, the first genetic map of persimmon was constructed and indicated that *D. oleifera* was one of the ancestors of cultivated persimmon. The persimmon genome represents an invaluable resource for the genetic improvement of the fruit and for better understanding of its genome evolution. Genetic markers can be developed based on this genome sequence, which will help to accelerate persimmon breeding.

## Materials and methods

### Genome size estimation

Young leaves of *D. oleifera* from three branches were used as the sample for genome sequencing (with each tree as one repetition). Nuclear DNA was isolated from fresh leaf tissue using a protocol developed by Doležel et al.^62^, and flow cytometry (BD FACScalibur, BD Biosciences, USA) was applied to estimate the nuclear DNA content. The reference standard was *Zea mays* ‘B73’ (2.35 pg/1C)^63^. The genome size of *D. oleifera* was calculated as the ratio between the sample and standard peaks multiplied by the genome size of the standard.

### De novo genome assembly and quality assessment

Mature leaves of *D. oleifera* ‘Youshi’ were collected from the National Persimmon Germplasm Resources Nursery (Northwest A&F University, Shaanxi, China), and the CTAB method^64^ was applied to extract the genomic DNA from the leaves. Library construction, sequencing, assembly and quality assessment for the ‘Youshi’ genome were conducted by Biomarker Technologies Corporation (Beijing, China). According to standard Illumina protocols, two paired-end (PE) libraries with insert sizes of 220 and 500 bp and 14 mate-pair (MP) libraries with insert sizes of 3, 4, 5, 8, 10, 15, and 17 kb were constructed. The Illumina HiSeq 2500 platform (Illumina, San Diego, CA, USA) was used to sequence these libraries and produced 88.35 Gb of raw sequencing data, representing 100X genome coverage. Reads were trimmed for adaptor removal and quality enhancement. NxTrim was used to collapse the duplicated reads of the MP libraries. A deduplication step was performed on the MP libraries.

The genome assembly and contigs were constructed and analyzed by ALLPATHS-LG^65^ with default parameters. SSPACE^66^ was used to link the contigs with support from the paired MP reads. Gaps were filled with GapCloser^67^. In preparation for the assembly, scaffolds <1000 bp were removed. PacBio library construction included DNA purification, damage repair, hairpin adaptor ligation, and digestion with exonucleases to remove damaged DNA and fragments without adaptors. The sizes of the templates (≥15 Kb) were selected via Blue Pippin electrophoresis, and the RS II platform (Pacific Biosciences, California, USA) using P6-C4 sequencing chemistry was then applied to sequence the library. The correction of the draft genome assembly was performed with the Canu v1.5^68^, WTDBG v1.2.8 (available at https://github.com/ruanjue/wtdbg) and Falcon^69^ (Supplementary Tables 31 and 32) assemblers. Finally, the assembly-merging method was utilized^70^ to optimize the assembly results.

Another assembly method for scaffold anchoring using Hi-C sequencing technology was applied. The gDNA was digested with *Hind*III. The 5′ overhangs were filled with biotinylated nucleotides; free blunt ends were ligated; and the DNA was purified and sheared to 300–700 bp fragments after biotin was removed. PCR was applied to enrich the biotin-tagged fragments, and the DNA was then used to construct an Illumina library, which was on the Illumina HiSeq 2500 platform. A total of 55.41 Gb of clean data (65 × the genome size) were obtained, and 62.20 M valid interaction pairs were detected to assist in genome assembly. The evaluation of the alignment efficiency and insert length distribution for valid pair fragments and valid interaction pairs was performed with HiC-Pro^71^. The genome sequence contigs and scaffolds were divided into subgroups and sorted and oriented into super scaffolds using LACHESIS^72^.

### Final assembly and validation

To improve the persimmon genome assembly and chromosome anchoring accuracy, the Illumina data, PacBio long reads and Hi-C data were combined and mutually corrected. Finally, the persimmon genome was assembled into 856.41 Mb of scaffold sequences. We found that 99.99% (856.35 Mb) of the genome consisted of the contig sequences. The assembly contained 4728 scaffolds, with a scaffold N50 of 42.42 Mb, composed of 5919 contigs, with a contig N50 of 890.84 kb. The maximum scaffold and contig lengths were 57.59 Mb and 9.38 Mb, respectively.

The Hi-C contact heatmap was inspected to confirm the accuracy of the Hi-C assembly. To facilitate the formation of Hi-C contacts, the sequences were proximate in three dimensions if they were adjacent to one another in one dimension. The number of Hi-C links between 100-kb windows on the pseudochromosomes of the final assembly is represented by the intensity of red coloration. More pronounced contact frequencies indicate more Hi-C links between the two bins. An elevated link frequency was observed with a diagonal pattern within individual pseudochromosomes, indicating increased interaction contacts between adjacent regions. The second-generation short sequencing reads from the RNAseq data were also used to validate the final assembly. Among the 250,587,312 reads, 98.60% were mapped successfully back to the final persimmon genome, with a 93.78% properly paired mapping rate.

CEGMA (version 2.5), with 248 conserved core eukaryotic genes, and BUSCO (version 2.0)^23^, with 1440 genes in embryophyta odb9, were used to assess the completeness and accuracy of the assembled persimmon genome.

### Genome annotation

A pipeline that integrated de novo gene prediction, homology-based gene models and RNAseq-based gene models was used to predict protein-coding genes. For de novo prediction, the genes in the genome were predicted with four predictors: Genscan^73^, Augustus (version 2.4)^74^, GlimmerHMM (version 3.0.4)^75^, GeneID (version 1.4)^76^ and SNAP^77^. For homology prediction, GeMoMa (version 1.3.1)^78^ was used, with protein sequences from *A. thaliana*, *O. sativa*, *Z. jujuba* and *C. sinensis* and gene models with a minimum 50% coverage and 50% query/hit coverage.

Two complementary methods, one de novo based and the other homology based, were used to identify and classify transposable elements (TEs). LTR FINDER (version 1.05)^79^, MITE-Hunter^80^, RepeatScout (version 1.0.5)^81^ and PILER-DF (version 2.4)^82^ were run to construct the de novo repeat libraries. RepeatMasker (version open-4.0.6)^83^ with WUBlast searches against the RepBase database (RepBase 19.06)^84^ was applied to build the homology-based repeat library. To obtain a consensus library, PASTEClassifer^85^ was used to process and classify the two libraries. To identify the TEs in the persimmon genome, RepeatMasker was employed, using the consensus library to mask the genome; the masked genome was used for gene prediction.

For RNA-seq prediction, processed reads were aligned to the reference genome using HISAT2^86^. HISAT (version 2.0.4)^87^ and Stringtie (version 1.2.3)^87^ were used to assemble the transcripts. Trinity (version 2.1.1)^88^ was applied for the de novo assembly of the RNA-seq reads, and the RNA-seq reads were analyzed with PASA (version 2.0.2)^89^ for the prediction of unigenes. TransDecoder (version 2.0) (Haas, http://transdecoder.github.io) and GeneMarkS-T (version 5.1)^90^ were used to predict open reading frames (ORFs). Finally, an integrated gene set was produced by EVM (version 1.1.1)^91^. The RNA-seq assemblies were employed to determine untranslated regions (UTRs). The longest transcripts for each locus were retained, and regions outside of the ORFs were designated as UTRs.

The Rfam database (version 12.0)^92^ and MirBase database^93^ were searched with Infernal (version 1.1)^94^ to detect microRNAs and rRNAs. tRNAscan-SE (version 1.3.1)^95^ with eukaryote parameters was applied to predict transfer RNAs (tRNAs).

GenBlastA (version 1.0.4)^96^ was used to identify homologous sequences in the genome by using the integrated gene set as the query, and GeneWise (version 2.4.1)^97^ was used to define pseudogenes containing premature stop codons or frameshift mutations with 60% identity and coverage.

Protein sequences were compared against several databases, including NR^98^, KOG^99^, KEGG^100^, and TrEMBL^101^, using BLAST (version 2.2.31)^102^ with an E-value cutoff of 1E-5 to annotate the functions of the predicted genes. GO annotations were assigned by BLAST2GO (version 2.5);^103^ InterProScan (version 5.8)^104^ and Hmmscan (HMMER, version 3.0)^105^ were used for searching; and annotatemotifs and domains were annotated by searching the Pfam database^106^, PROSITE database^107^ and HAMAP database^108^.

### Comparative genomic analysis

The available protein sequence sets were collected from 14 sequenced plant species: *Vitis vinifera* (Vvinifera_145_Genoscope), *Actinidia chinensis* (v1.0), *Musa acuminata*, *Solanum lycopersicum* (SL2.40), *Diospyros oleifera*, *Citrus sinensis*, *Glycine max* (Soybean_Williams82), *Carica papaya*, *Arabidopsis thaliana* (TAIR10), *Juglans regia*, *Fragaria vesca*, *Prunus persica* (v2.0), *Pyrus bretschneideri*, and *Malus domestica* (v1.0). OrthoMCL (version 2.0; mcl inflation factor of 1.5)^109^ was used to identify orthologous genes. Pairwise sequence similarities were calculated by all-against-all BLASTP (Blast + version 2.3.0)^110^ with a *P*-value cutoff of 1e−5 and a minimum match length of 50%.

MUSCLE^111^ was used to perform the phylogenetic analysis and alignment using the coding sequences of common single-copy genes. The jModelTest output was used to select the protein model, and the evolutionary tree was constructed via the TIM2+I+G model. The divergence time was estimated with MCMCTREE of PAML (version 4.7a)^112^. Gene family expansion and contraction were analyzed by using CAFÉ (version 4.2)^113^ with a maximum-likelihood model. The gene family clustering results and estimated divergence times between species were used. The value of the birth and death parameter (λ) was 0.02, and the *P*-value was 0.01.

MCscan was applied to detect the 4DTV gene pairs calculated using the HKY substitution model. MCscan with the YN00 program of the PAML package was used to detect the synonymous mutation rate (ks) of gene pairs.

### Persimmon fruit deastringency treatment and transcriptome sequencing

Commercially mature persimmon fruit from 53 astringent types were harvested from the National Persimmon Germplasm Resources Nursery (Northwest A&F University, Shaanxi, China) in 2017. To remove the astringency from mature fruit, high CO_2_ (95% CO_2_ + 4% N_2_ + 1% O_2_, 1 d) treatment was applied as described by Zhu et al.^17,18^, and fruit treated in air were used as the control (CK). The treatments were carried out with three biological replicates. At each sampling time, flesh samples were bulked and frozen in liquid nitrogen and stored at −80 °C for further use.

Fruit astringency was evaluated by two different methods. First, soluble tannins were visualized via the tannin printing method according to Min et al.^114^. The fruit were cut lengthwise immediately after treatment and then pressed onto 5% FeCl_2_-soaked filter paper for 5 s. The intensity of the resulting black color after the removal of fruit indicated the soluble tannin content, and the filter paper was photographed. The soluble tannin contents of frozen samples were also quantified with the Folin-Ciocalteau reagent according to the method described by Yin et al.^115^. The results were calculated using a standard curve of tannin acid equivalents g^−1^ fresh weight. All measurements were conducted with three biological replicates.

Four cultivars showing obvious differences in the rates of deastringency (e.g., rapid deastringency, Sigoushi and Luoyangfangtianshengshi; slow deastringency, Laopige, and Shijiazhuanglianhuashi) were selected for RNA-seq analysis. A total of 36 samples (0 d, 1 d for CK and CO_2_-treated fruit) with three biological replicates were used for total RNA extraction via the CTAB protocol described by Chang et al.^64^. An Agilent 2100 Bioanalyzer (Agilent Technologies, Santa Cara, California, USA), a NanoDrop 2000 spectrophotometer (Thermo Scientific, Waltham, MA, USA), and a Qubit 2.0 fluorometer (ThermoFisher Scientific, Waltham, MA, US) were used to assess RNA integrity, purity, and concentrations, respectively. A TruSeq RNA Sample Preparation Kit was used to prepare RNA-seq libraries following the manufacturer’s instructions, and the libraries were sequenced on the Illumina HiSeq 2500 platform (Illumina, San Diego, CA, USA). Raw reads were trimmed to remove adaptors and increase quality. Reads <100 bp were discarded after trimming. Overall, each sample produced an average of 7.28 Gb clean data.

The clean reads were mapped to the genome by using HISAT2^86^ with the default parameters. StringTie^116^ was used to assemble the transcripts. The fragments per kilobase of transcript per million fragments mapped (FPKM) values were used to measure gene expression. DEseq^117^ was applied to identify differentially expressed genes (DEGs). The false discovery rate was used to adjust the *P*-values. Genes with significant differences in expression (i.e., log_2_ foldchange >1 and adjusted *P*-value < 0.01) were considered DEGs and were annotated with GO terms and KEGG pathways.

Coexpression networks were constructed using the WGCNA (v1.66) package in R (v3.5.2)^118^. Genes with expression values (FPKM) in any variable equal to or higher than 1 were selected for WGCNA. The automatic network construction function *blockwiseModules ()* with the following settings: soft power 16, minModulesSize 30, mergeCutHeight 0.25 and default parameters for other settings, was used to construct the models. The soft power was chosen by the function *pickSoftThreshold ()* in WGCNA. The correlations between module eigengenes, the first principle of each module, and the content of tannins were calculated with function *cor ()* in R with default settings. Candidate hub genes in “tan”, “brown” and “yellow” were picked by thresholding at a value of 0.6. The coexpression network of 295 selected genes was visualized with Cytoscape (v3.7.1).

### Real-time PCR

For real-time PCR, gene-specific oligonucleotide primers were designed, which are described in Supplementary Table 32. Melting curves and product resequencing were used to assess the quality and specificity of each pair of primers. Real-time PCR was carried out on a Bio-Rad CFX96 Real-Time PCR System using SsoFast^TM^ EvaGreen Supermix (Bio-Rad, USA) following the manufacturer’s instructions. The housekeeping gene *DkACT* (GenBank No. AB473616)^15^ was chosen as the internal control, and the 2^−ΔCt^ method was used to calculate the relative expression^119^.

### Dual-luciferase assay

The in vivo regulatory effects of the previously characterized high-CO_2_/hypoxia-responsive transcription factors on the newly identified deastringency-related gene (*DkPK1*) were investigated via dual-luciferase assays. The promoter of *DkPK1* was cloned into the pGreen II 0800-LUC vector (LUC) (primers are listed in Supplementary Table 28), and the transcription factors were previously cloned into the pGreen II 002962-SK vector^17,18^. All constructs were electroporated into *Agrobacterium tumefaciens* GV1301. The constructed SK and LUC plasmids were transiently expressed in tobacco (*Nicotiana benthamiana*) leaves as described by Min et al.^9^. The Dual-luciferase® Reporter Assay System 10-Pack kit (Promega, USA) was employed to analyze firefly luciferase and *Renilla* luciferase in tobacco leaves at 3 d after infiltration by using a GLOMAX^TM^ 96 Microplate Luminometer (Promega).

### SLAF library construction and high-throughput sequencing

As mentioned above, it is difficult to obtain hybrid persimmon offspring due to the abortion of hybrid embryos in seeds. In this study, a population of 77 F1 plants derived from ‘Luotiantianshi’ × ‘Taishuu’ via the embryo rescue culture technique was used to construct the *Diospyros kaki* (2n = 6x = 90) genetic map. Young leaves were collected for DNA extraction. The cetyltrimethylammonium bromide method^120^ was used to extract the total genomic DNA. DNA concentrations and qualities were estimated using a NanoDrop 2000 spectrophotometer (Thermo Scientific, Milan, Italy) and electrophoresis in agarose gels.

SLAF-seq was used for the rapid and effective discovery of SNP markers, employing an improved SLAF-seq strategy as described by Sun et al.^57^. Two restriction enzymes (i.e., *Rsa*I and *Hae*III) were tested to identify the best enzyme for GBS (genotyping by sequencing) library construction by investigating the length and distribution of the resulting digested fragments of the genomic DNA of the two parents and the F1 population. Fragments of 314–414 bp were selected to generate paired-end reads (PE125 bp) on the Illumina HiSeq-Xten sequencing platform (Illumina, San Diego, CA, USA) by Biomarker Technologies, Beijing, China.

### Genotyping and linkage map construction

SLAF marker identification and genotyping were performed using procedures described by Sun et al.^57^. Briefly, low-quality reads (quality score <20) were filtered out, and then filtered reads were clustered according to sequence similarity. Identical reads were merged to avoid repeat-computing requirements, and sequences with over 90% identity were grouped into a single SLAF locus, as described by Sun et al.^57^. Differences in high-depth fragments were defined as SNPs or indels. To construct a high-quality genetic map, we filtered the SLAFs using five criteria: (1) removal of SLAFs from parents where the sequencing depth was less than 10X ; (2) removal of SLAFs with more than three SNPs; (3) removal of SLAFs with an aa × bb segregation pattern; (4) removal of SLAFs missing more than 30% of offspring; and (5) removal of segregation-distorted markers (*p* < 0.01). A high-density genetic map was constructed by using the SLAFs that passed the five-step filtering process. SLAFs that followed the 1:1 Mendelian segregation pattern were used for genetic map construction.

The map was constructed with HighMap software^121^, which performs four procedures: module-linkage grouping, marker ordering, error genotyping correction, and map evaluation. The single-linkage clustering algorithm was used to cluster the markers into linkage groups. The error correction strategy by using SMOOTH was then conducted according to the parental contribution of genotypes^122^, and a k-nearest neighbor algorithm was applied to impute missing genotypes^123^. Skewed markers were then added to this map by applying a multipoint method of maximum likelihood. Map distances were estimated using the Kosambi mapping function^124^. Based on the *D. oleifera* genome, the SLAF sequences of the 11,204 mapped markers were aligned to the *D. oleifera* genome using BLASTN analysis with an e-value cutoff of 1e−5.

## Accession numbers

The raw genomic sequence, transcriptome, and genetic map data have been deposited in the NCBI Sequence Read Archive under accession numbers PRJNA562043, PRJNA562975, and PRJNA563228.

## Supplementary information

Supplemental figures

Supplemental Tables
