# Supplementary material for: The persimmon (Diospyros oleifera Cheng) genome provides new insights into the inheritance of astringency and ancestral evolution
Source: Hortic Res. 2019 Dec 18;6:138. doi: 10.1038/s41438-019-0227-2 (PMC6917749; doi:10.1038/s41438-019-0227-2)

Supplemental Figure 1 The fruit of ‘Youshi’ persimmon (*Diospyros oleifera* Cheng)


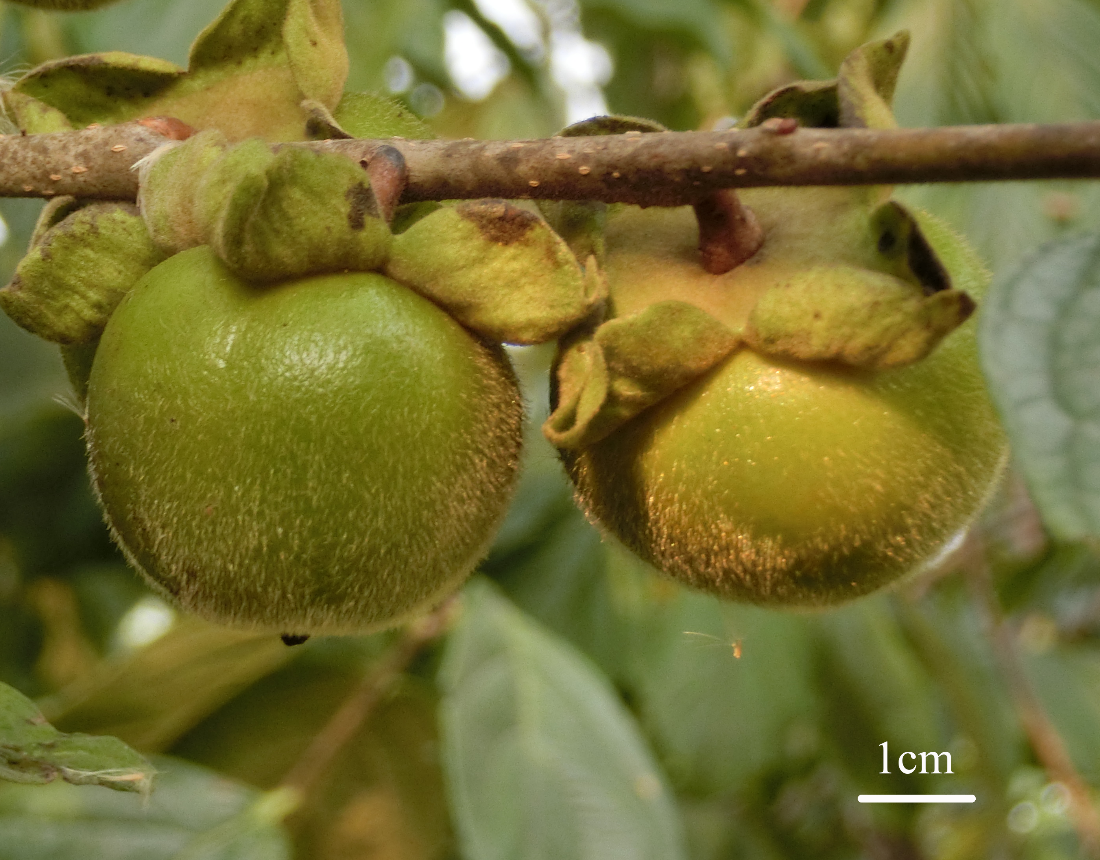


Supplemental Figure 2 The assembly flow diagram of the *D. oleifera* genome.


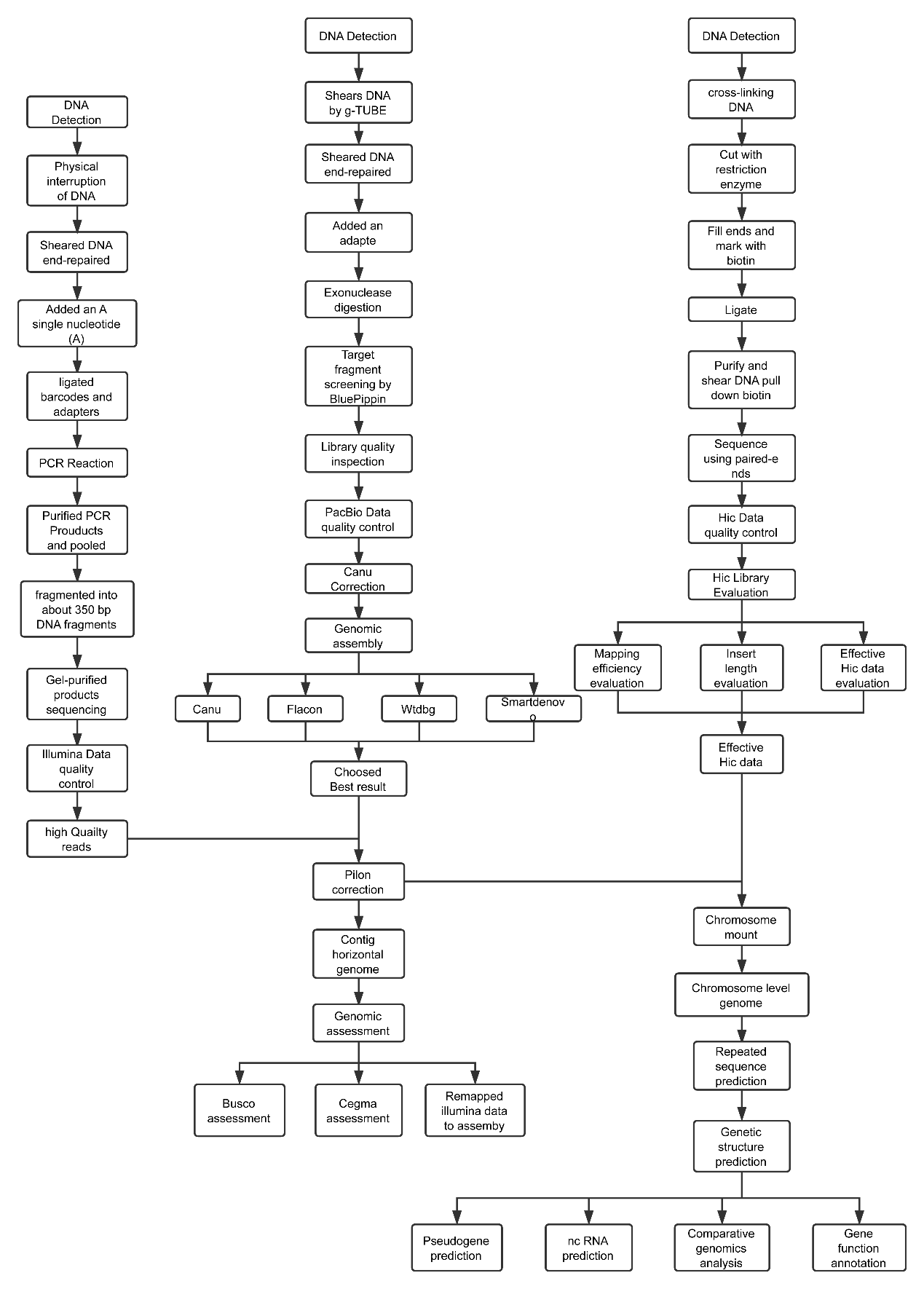


Supplemental Figure 3 Estimate of genome size from nuclear weight measured by flow cytometry.


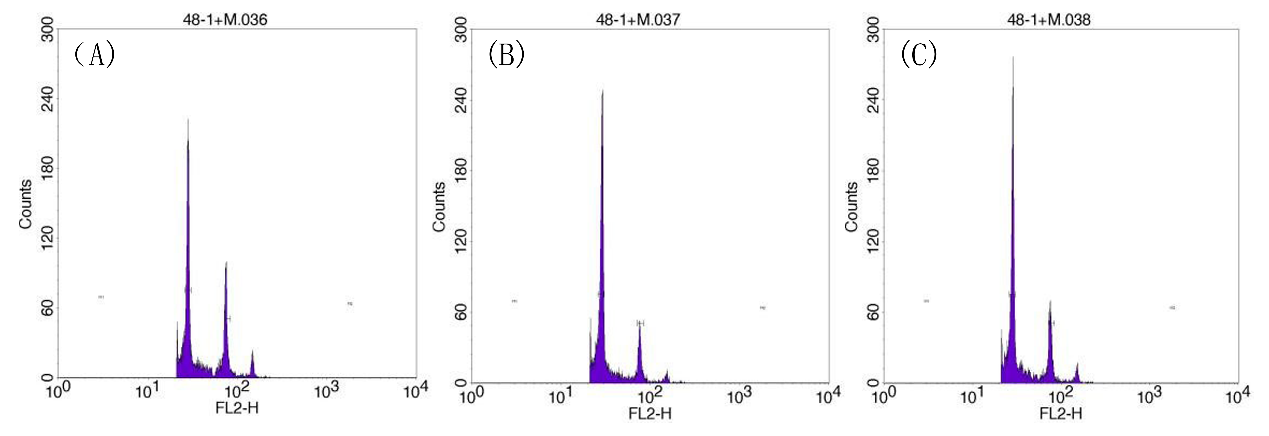


Supplemental Figure 4 Heatmap of Hi-C assembly chromosome interaction


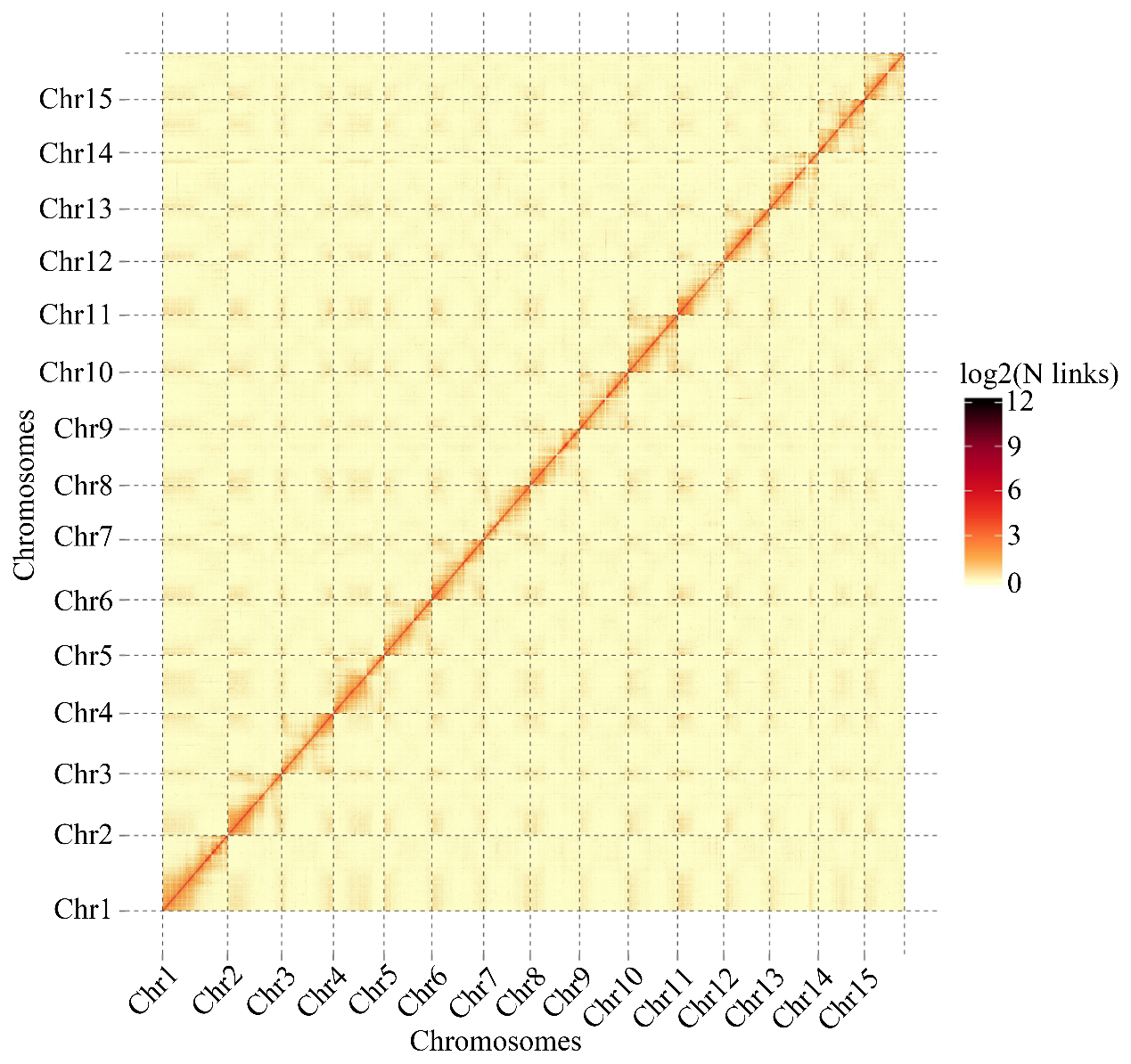


Supplemental Figure 5 Distribution map of genes from the three prediction methods


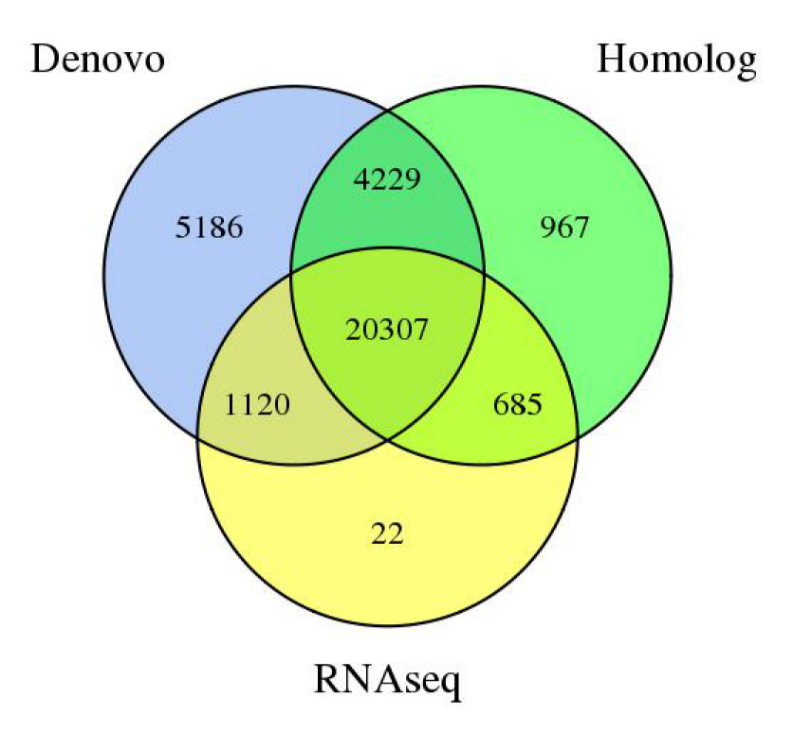


Supplemental Figure 6 Density of genes anchored to the 15 pseudochromosomes


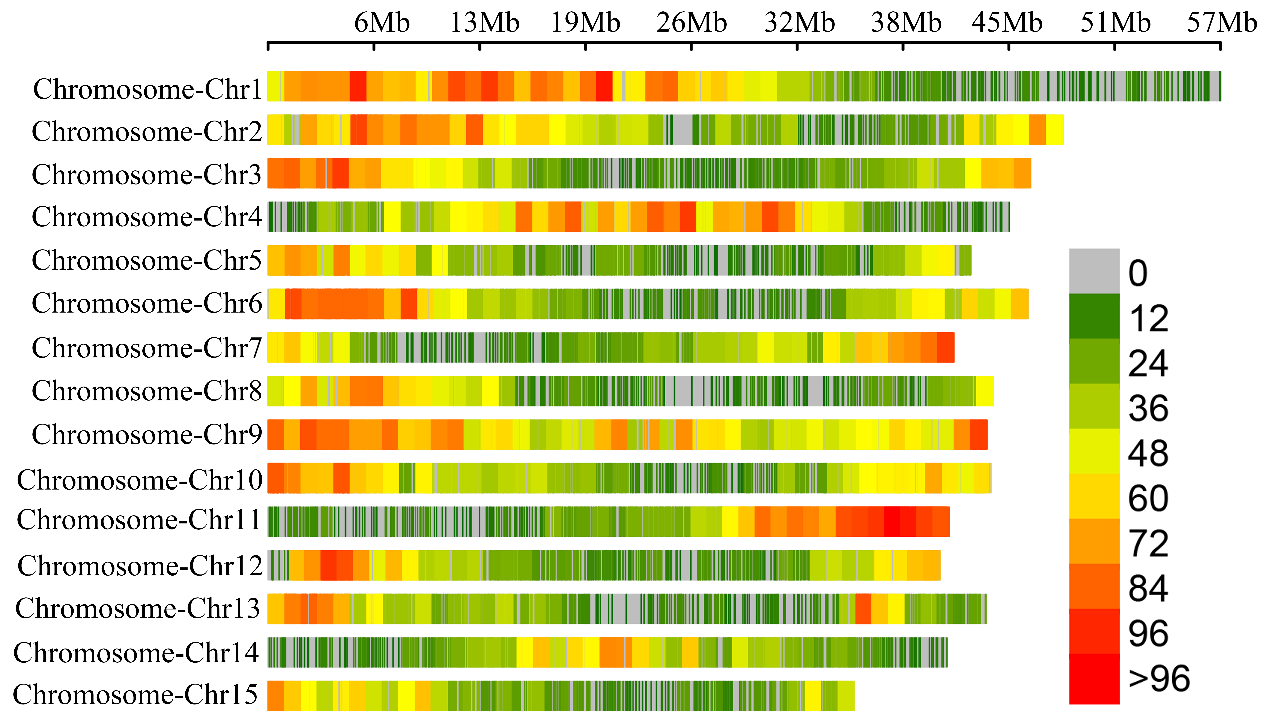


Supplemental Figure 7 Ks analysis of the persimmon genome with three other genomes

.


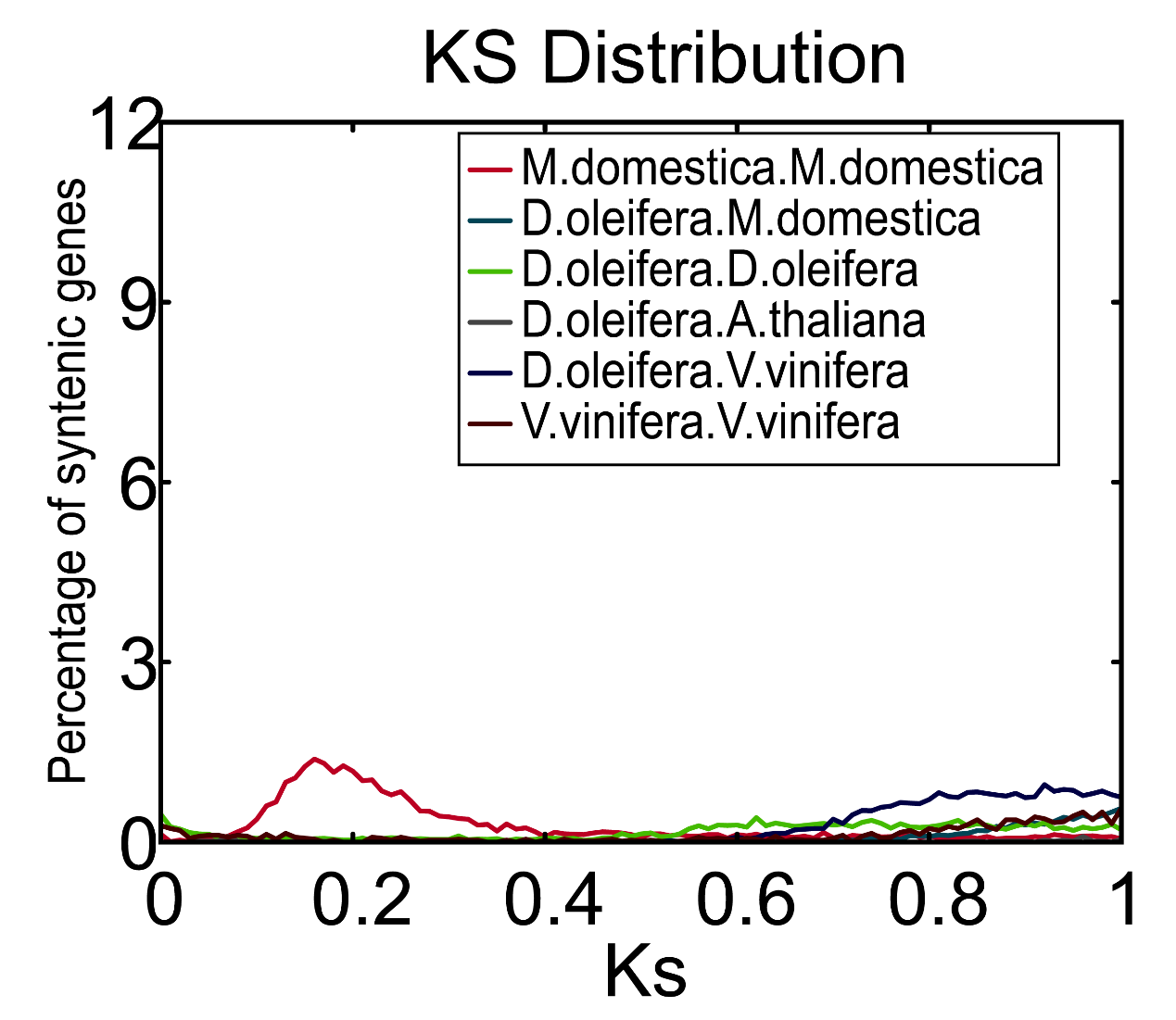


Supplemental Figure 8 Detailed gene cluster information for PA biosynthesis genes on chromosome 1


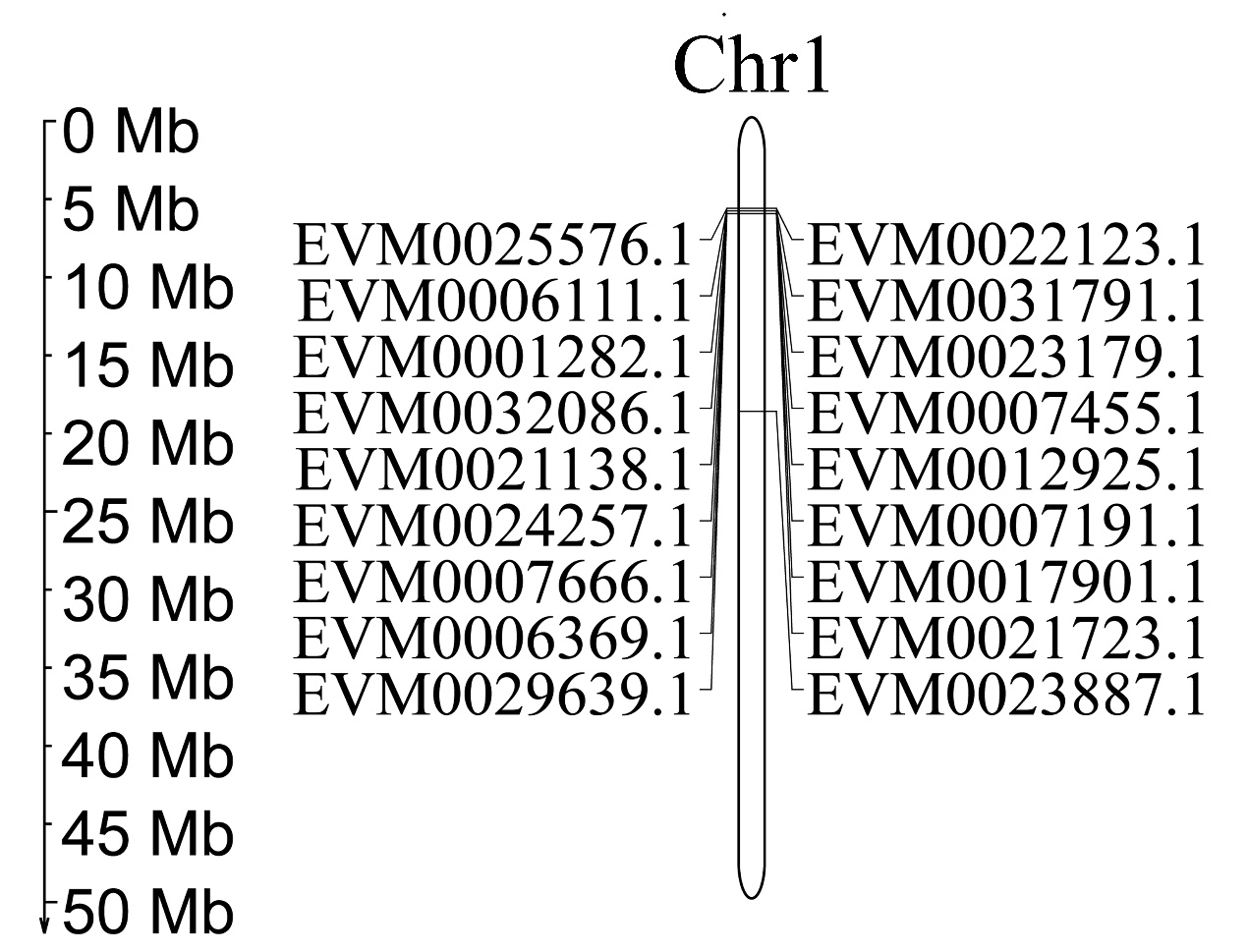


Supplemental Figure 9 Pathway of acetaldehyde synthesis under low oxygen


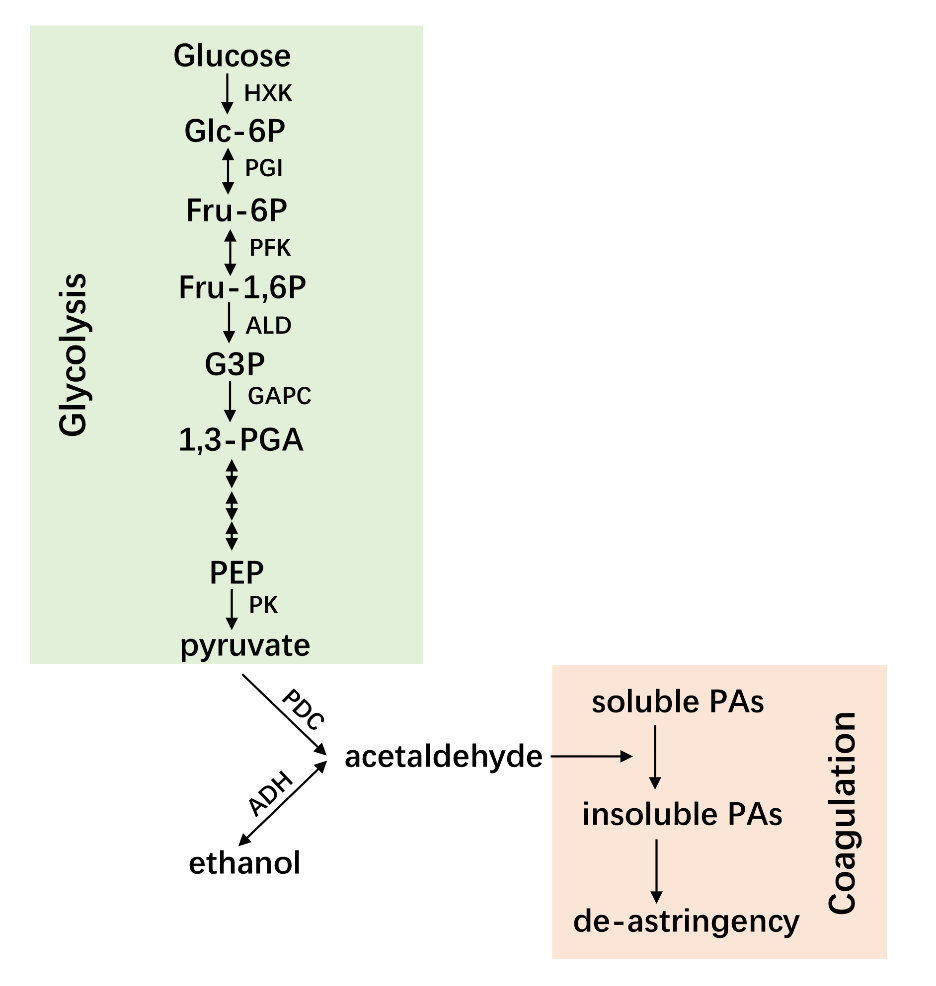


Supplemental Figure 10 Hub-gene network of the brown, the yellow and tan modules. The size of the dots represents hubness. The candidate genes are shown in green, yellow and brown colors circles. Red highlights the *DkPK1*, *DkPDC1* and *DkPDC2*.


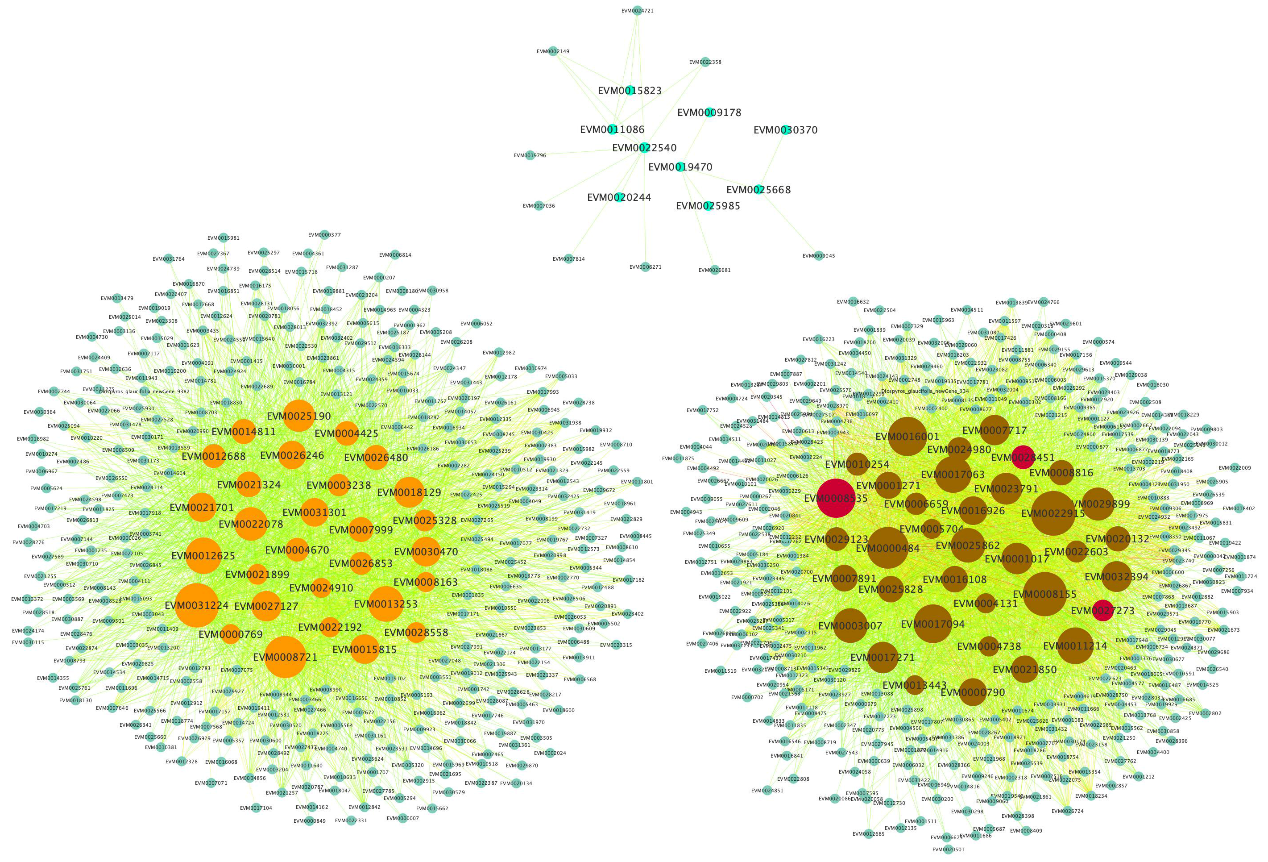


Supplemental Figure 11 Low-oxygen-responsive motif analysis in the promoters of previously identified key transcription factors involved in persimmon fruit deastringency.


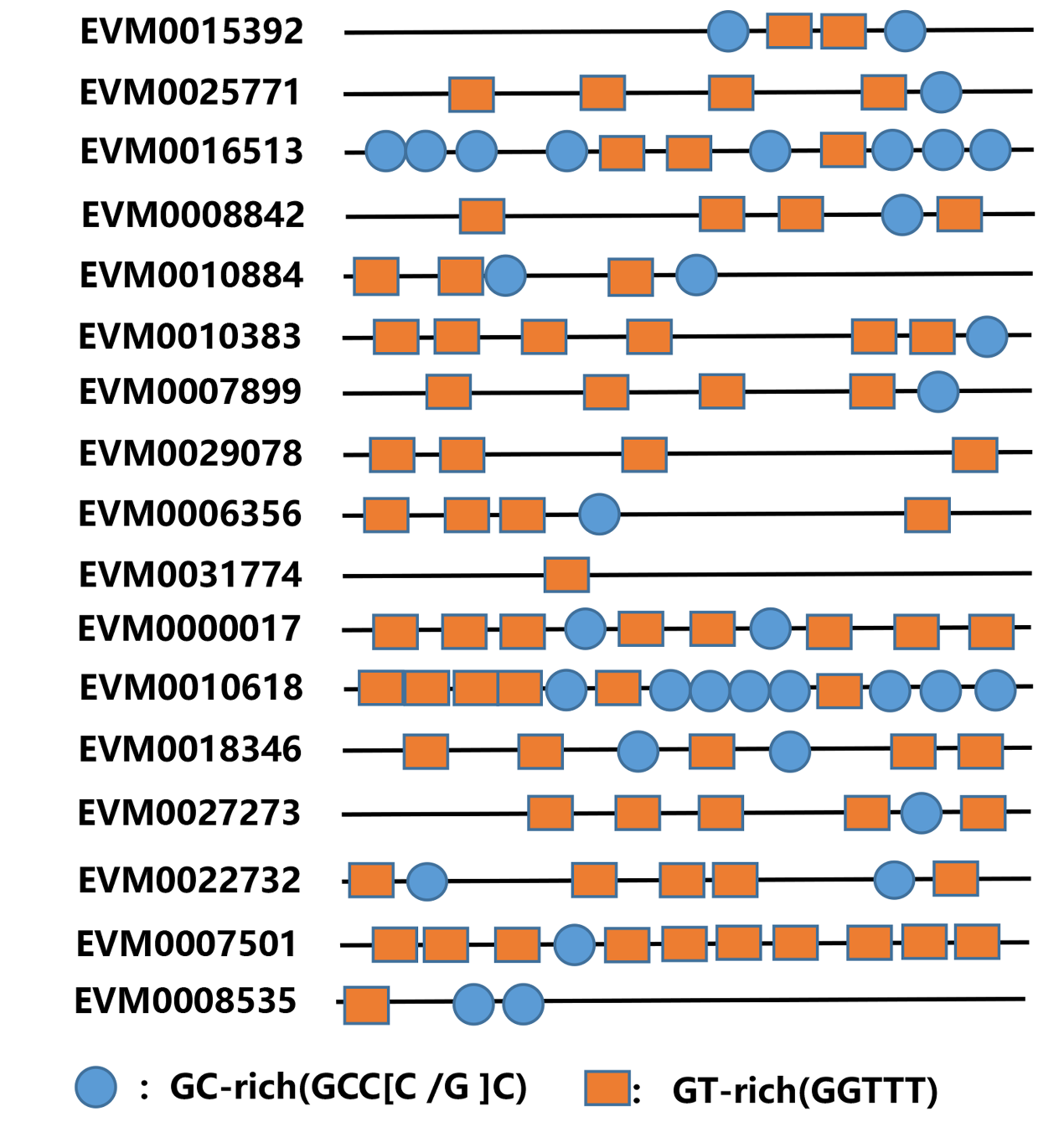


Supplemental Figure 12 Low-oxygen-responsive motif analysis in the promoters of target genes.


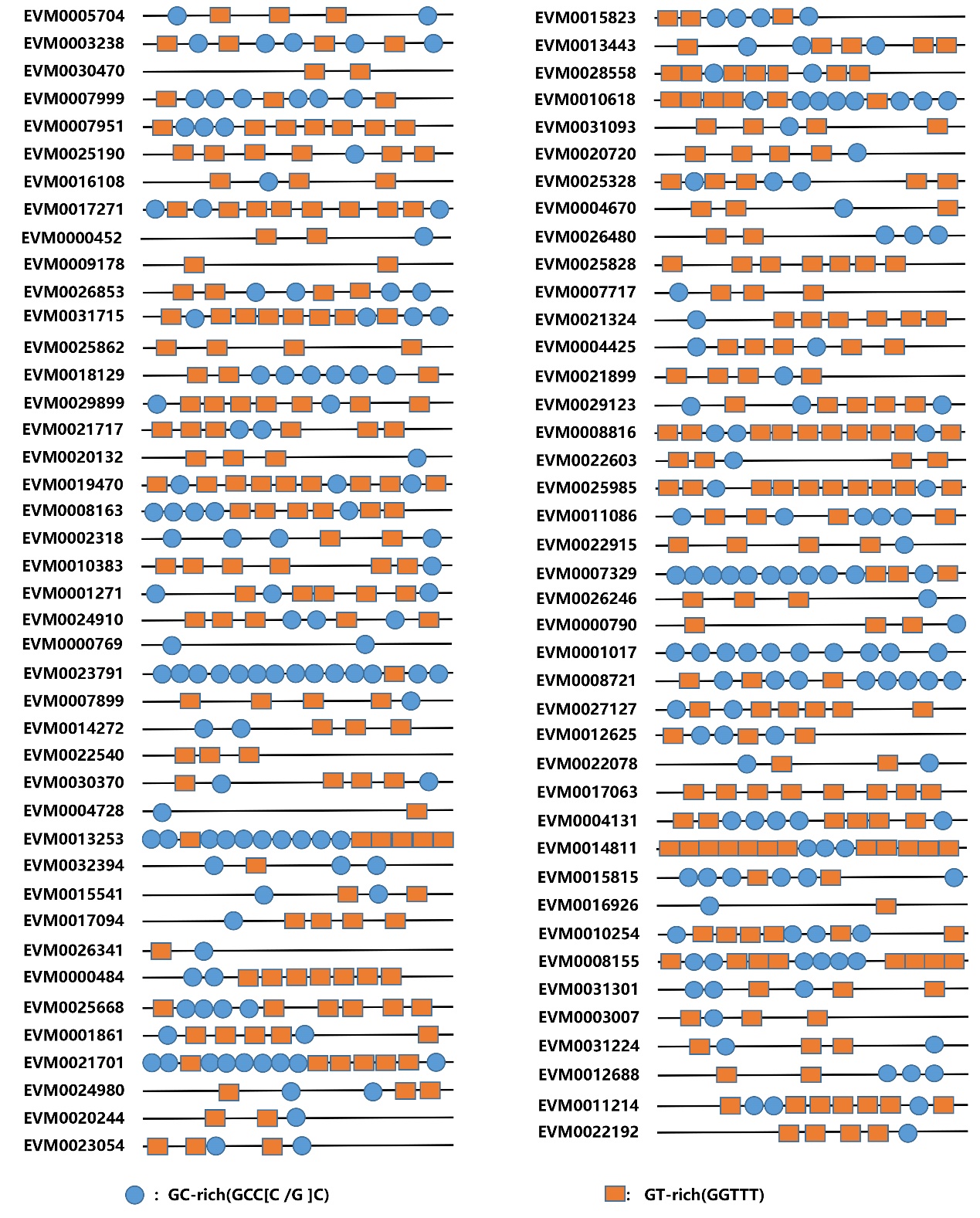


Supplemental Figure 13 *D.kaki* genetic map validation by haplotype mapping. Each two rows represent a genome in an F1 population including 76 progenies and 2 parents. Columns correspond to LG. Green and blue shading indicate paternal or maternal haplotypes, and grey shading indicates missing data. Only fewer markers were found in small recombination blocks.


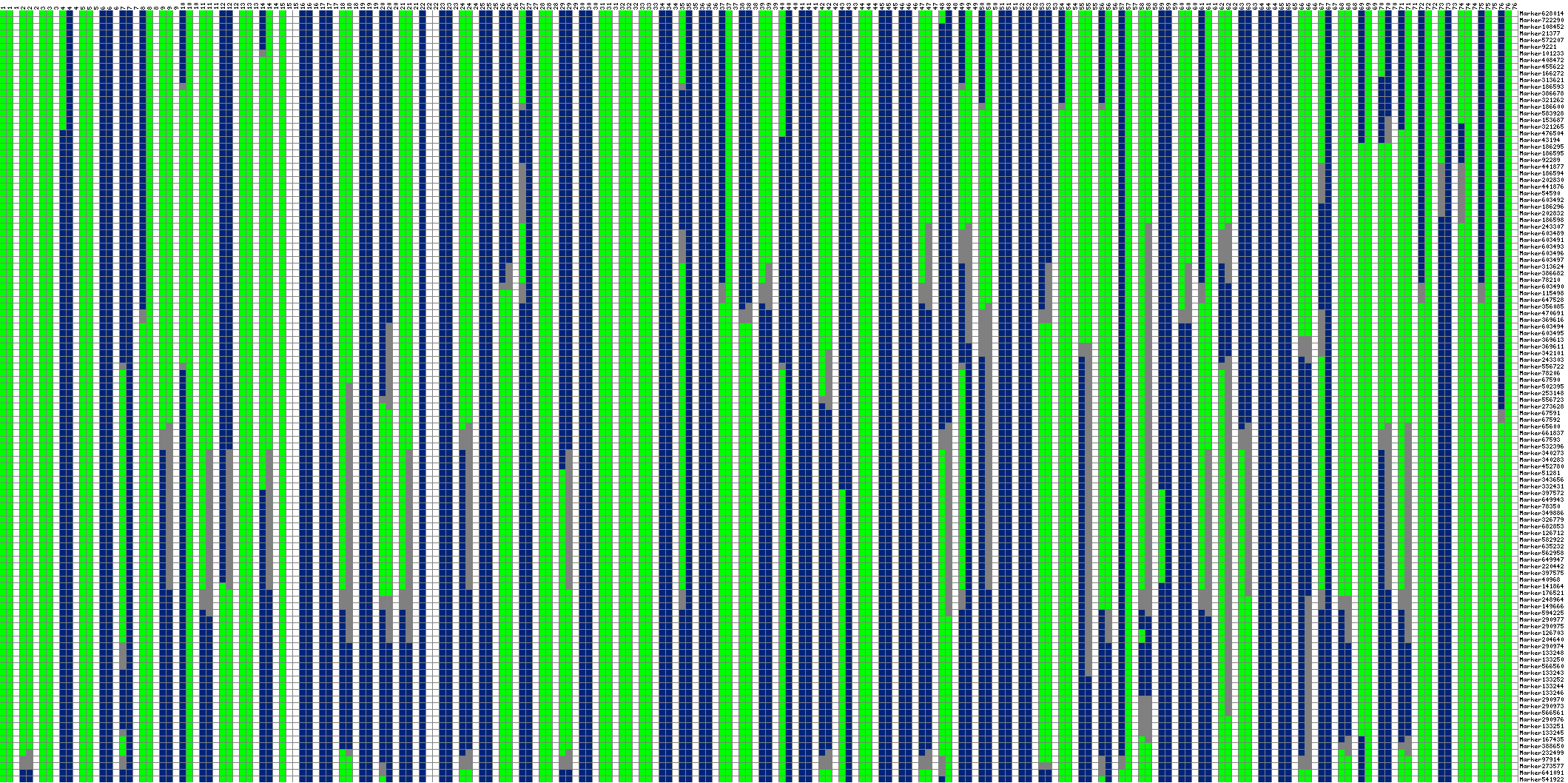


Supplemental Figure 14 Heatmaps of the *D. kaki* genetic map. The distribution of the mapped markers is shown as small squares based on the order in the *D. kaki* genetic map. The color of every small square represents recombination rate between two mapped markers. The change of color from yellow to purple represents the change of recombination rate from small to large.


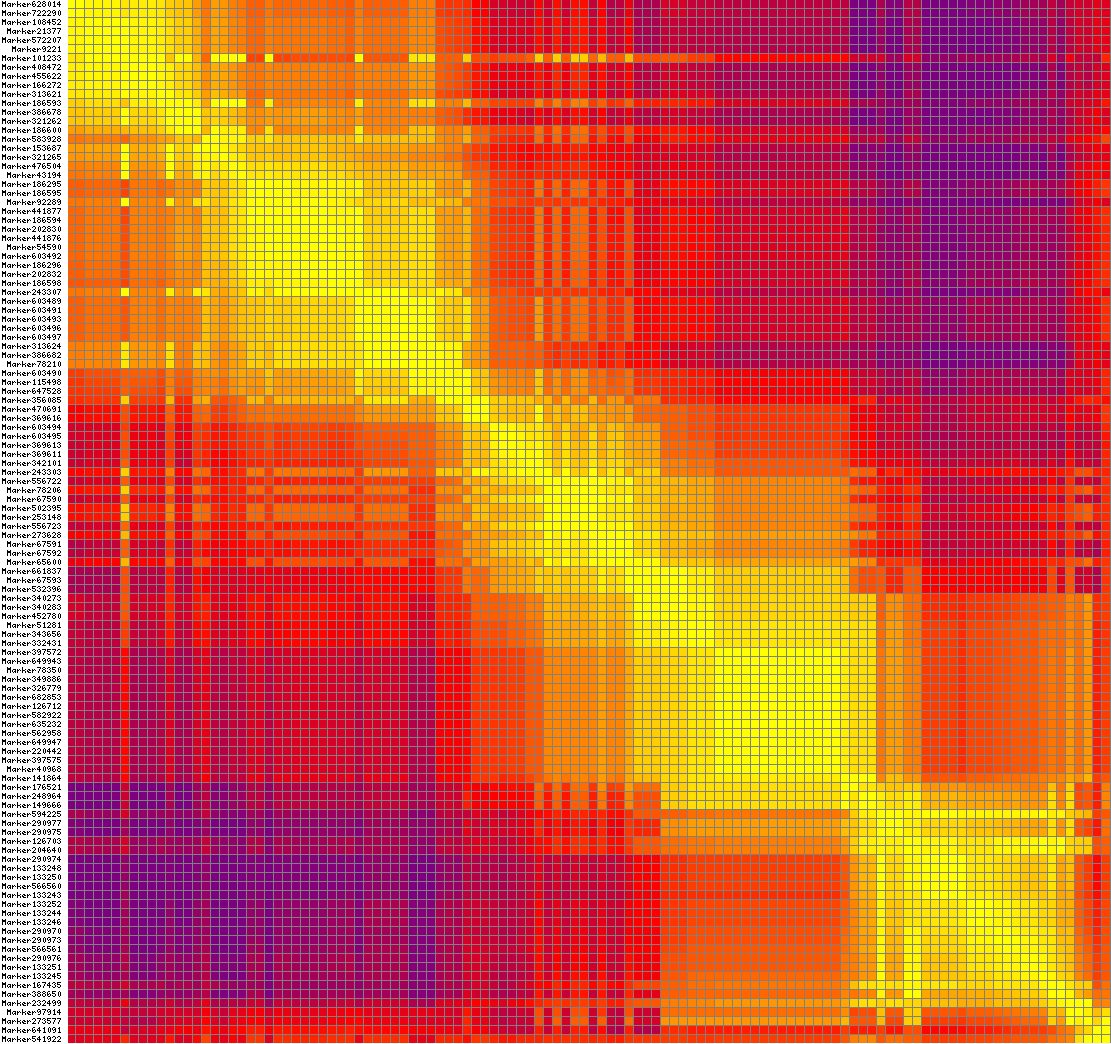

Supplement: Supplementary file 1 — Supplemental figures [file 41438_2019_227_MOESM1_ESM.docx]
